# An Artificial Intelligence–Based Framework for Predicting Emergency Department Overcrowding: Development and Evaluation Study

**DOI:** 10.2196/73960

**Published:** 2025-09-17

**Authors:** Orhun Vural, Bunyamin Ozaydin, Khalid Y Aram, James Booth, Brittany Freeman Lindsey, Abdulaziz Ahmed

**Affiliations:** 1 Department of Electrical and Computer Engineering School of Engineering University of Alabama at Birmingham Birmingham, AL United States; 2 Department of Health Services Administration School of Health Professions University of Alabama at Birmingham Birmingham, AL United States; 3 Department of Biomedical Informatics and Data Science Heersink School of Medicine University of Alabama at Birmingham Birmingham, AL United States; 4 School of Business & Technology Emporia State University Emporia United States; 5 Department of Emergency Medicine Heersink School of Medicine University of Alabama at Birmingham Birmingham, AL United States; 6 Department of Patient Throughput University of Alabama at Birmingham Hospital Birmingham, AL United States

**Keywords:** emergency overcrowding, time series prediction, patient flow forecasting, hospital resource management, reactive and proactive full capacity protocol

## Abstract

**Background:**

Emergency department (ED) overcrowding remains a critical challenge, leading to delays in patient care and increased operational strain. Current hospital management strategies often rely on reactive decision-making, addressing congestion only after it occurs. However, effective patient flow management requires early identification of overcrowding risks to allow timely interventions. Machine learning (ML)–based predictive modeling offers a solution by forecasting key patient flow measures, such as waiting count, enabling proactive resource allocation and improved hospital efficiency.

**Objective:**

The aim of this study is to develop ML models that predict ED waiting room occupancy (waiting count) at 2 temporal resolutions. The first approach is the hourly prediction model, which estimates the waiting count exactly 6 hours ahead at each prediction time (eg, a 1 PM prediction forecasts 7 PM). The second approach is the daily prediction model, which forecasts the average waiting count for the next 24-hour period (eg, a 5 PM prediction estimates the following day’s average). These predictive tools support resource allocation and help mitigate overcrowding by enabling proactive interventions before congestion occurs.

**Methods:**

Data from a partner hospital’s ED in the southeastern United States were used, integrating internal and external sources. Eleven different ML algorithms, ranging from traditional approaches to deep learning architectures, were systematically trained and evaluated on both hourly and daily predictions to determine the models that achieved the lowest prediction error. Experiments optimized feature combinations, and the best models were tested under high patient volume and across different hours to assess temporal accuracy.

**Results:**

The best hourly prediction performance was achieved by time series vision transformer plus (TSiTPlus) with a mean absolute error (MAE) of 4.19 and a mean squared error (MSE) of 29.36. The overall hourly waiting count had a mean of 18.11 and a SD (σ) of 9.77. Prediction accuracy varied by time of day, with the lowest MAE at 11 PM (2.45) and the highest at 8 PM (5.45). Extreme case analysis at (mean + 1σ), (mean + 2σ), and (mean + 3σ) resulted in MAEs of 6.16, 10.16, and 15.59, respectively. For daily predictions, an explainable convolutional neural network plus (XCMPlus) achieved the best results with an MAE of 2.00 and a MSE of 6.64. The daily waiting count had a mean of 18.11 and a SD of 4.51. Both models outperformed traditional forecasting approaches across multiple evaluation metrics.

**Conclusions:**

The proposed prediction models effectively forecast ED waiting count at both hourly and daily intervals. The results demonstrate the value of integrating diverse data sources and applying advanced modeling techniques to support proactive resource allocation decisions. The implementation of these forecasting tools within hospital management systems has the potential to improve patient flow and reduce overcrowding in emergency care settings. The code is available in our GitHub repository.

## Introduction

### Background

Emergency departments (EDs) are the primary source of hospital admissions, even though most individuals seen in the ED are ultimately discharged [1]. In 2021, there were more than 140 million visits to American EDs [2], of which 14.5 million (10.4%) led to hospital inpatient admissions, and 2 million (1.4%) led to admission to critical care units [3]. Several factors lead to ED overcrowding, including complexity of complaints and injuries [4,5], resource limitations [6], large patient volumes [7], and inefficient flow of patients [8]. ED overcrowding is associated with poor health care outcomes. It can lead to delays in diagnosis and treatment, which can result in poorer patient outcomes, higher comorbidities, and increased patient illness [9,10]. For instance, it was found that patients with acute coronary syndrome who presented to overcrowded EDs had a significantly higher rate of serious complications compared with those who presented to noncrowded EDs (6% vs 3%). The complications include death, late myocardial infarction, cardiac arrest, arrhythmias, heart failure, stroke, and hypotension [11]. Treatment delays also lead to serious complications, including death [12]. Sprivulis et al [13] observed a significant linear relationship between ED overcrowding and patient mortality based on 3 years of data from 3 large hospital systems.

The Emergency Medicine Practice Committee of the American College of Emergency Physicians reported that ED overcrowding is a hospital-wide patient flow problem rather than an isolated ED problem [1,2]. The full capacity protocol (FCP), a key approach recommended by the American College of Emergency Physicians to improve patient flow across the entire hospital, is an internationally recognized communication tool between the ED and inpatient units [8]. It contains a set of interventions that can be tailored to the severity levels of ED overcrowding [14]. A set of criteria triggers each intervention level. The criteria are based on different patient flow measures (PFMs), such as the number of patients waiting to be admitted to an inpatient unit (boarded patients) [14,15].

In the current FCP practice, the unit responsible for managing patient flow relies on near real-time PFM values to activate different FCP levels, which is a reactive approach. Specifically, at our partner hospital—an urban, university-affiliated academic medical center in the southeastern United States—this unit is referred to as the patient flow coordination team (PFCT). Implementing FCP interventions (eg, creating hallway treatment spaces or activating on-call personnel) requires preparation time, forcing PFCT to prepare and act simultaneously when the ED is already overcrowded, significantly increasing their stress. Therefore, accessing the predicted PFM values before overcrowding can provide the PFCT enough time to prepare before they implement FCP interventions. Given the critical role of timely and accurate information in driving FCP interventions, it is essential to develop prediction models to forecast PFMs used by the FCP criteria, transforming FCP from reactive to proactive. Proactive FCP can help in planning and implementing interventions at different crowding severity levels proactively before the ED is already overcrowded. Predicted PFMs built into a proactive FCP can help the PFCT anticipate future FCP level escalation and prepare for interventions, such as coordinating staffing needs and creating additional hallway treatment spaces.

There are many PFMs that can be used to determine the FCP level, such as ED hourly waiting count, ED boarding count, ED arrival count, and the number of patients by Emergency Severity Index (ESI), among others. For this study, the advisory board of the project recommended that we start with building a prediction model for the ED waiting count, as it is one of the most important PFMs. The ED waiting count represents the number of patients who have arrived at the ED but have not yet been moved to a treatment room or started their clinical care. The advisory board members represent different ED units at the partner hospital. The members include the chair of Emergency Medicine, the chief medical information officer, the associate principals of the Office of Clinical Practice Transformation, the associate vice president of clinical operations at the center of patient flow, the senior director of Emergency Services, and the nurse director for Emergency Services. Based on the feedback from the advisory board, the goal of this paper is to build deep learning models to predict the ED waiting count at two time points: (1) In the hourly basis, in which the prediction models forecast the waiting count in the next 6 hours; (2) In daily basis where the models predict the average waiting count for next working day. The prediction information allows PFCT to prepare ED resources to improve patient flow and consequently mitigate ED overcrowding. This study is part of a larger funded ED improvement project, and the models presented in this study will be integrated into a decision support system to be used by our partner hospital.

As part of a broader effort to build a data-driven decision support system for ED operations, this paper focuses on the critical metric of ED waiting room count. This predictive model represents a foundational component of the broader effort to operationalize proactive FCP. Using real-world operational and contextual data, we assess the utility of multiple time-series algorithms and dataset variations to inform timely, data-driven predictions in ED management. The contributions of this study are as follows:

- We proposed an improvement for the current reactive FCP to make it proactive based on prediction models.
- This study was based on a real-world dataset and was conducted in collaboration with key decision makers from the ED.
- We developed predictive models that integrate multiple predictors from various sources to provide a comprehensive understanding of patient flow dynamics and improve forecasting performance.
- We introduced 2 complementary prediction approaches—hourly and daily—that provide actionable insights at different time scales.

### Prior Work

Numerous studies in the literature have focused on predicting different ED PFMs or outcomes. Traditional models have been developed to predict hospital admissions at ED triage, often relying on a limited set of demographic, administrative, and clinical variables [16-18]. For example, Parker et al [17] used variables such as age group, race, postal code, day of the week, time of day, triage category, mode of arrival, and fever status in a logistic regression model to predict hospital admissions with an area under the curve of 0.825. Similarly, Sun et al [18] developed a model to predict immediate hospital admission at the time of ED triage using routine administrative data, including age, patient acuity category, arrival mode, and coexisting chronic diseases like diabetes, hypertension, and dyslipidemia, achieving an area under the curve of 0.849. However, because these models focus on only individual patient-level triage outcome predictions, they offer limited utility for administrative management in addressing ED crowding, as they do not provide a comprehensive view of overall patient flow or resource utilization.

In one of the earlier studies that focused on predicting ED PFMs at the aggregate patient level, Schweigler et al [19] developed a baseline model to predict ED overcrowding using historical averages and compared its performance to more advanced time series models, including seasonal autoregressive integrated moving average (ARIMA) and a sinusoidal model with an autoregressive error term. However, these models relied solely on historical averages to predict ED crowding based on bed occupancy, without considering any other predictors, limiting their utility for comprehensive ED crowding management. A subsequent time series modeling study by Kadri et al [20] also developed an ARIMA model to predict daily pediatric ED attendances using only historical attendance data, without external predictors.

Recent studies have leveraged advanced machine learning (ML) models to predict ED PFMs, which are generally the number of patients arriving [21-23] or waiting time [24,25], across different prediction horizons. Our study targets waiting count prediction, which measures the number of patients present in the ED waiting room during each time interval by cumulatively adding patients for every hour they remain. Arrival count and waiting count are fundamentally different PFMs: arrival count records each patient once, at the moment they enter the ED, whereas waiting count tracks each patient for every hour they remain in the waiting area. For example, if a patient arrives at 1:05 PM and leaves at 3:35 PM, they are counted for 1-2 PM in the arrival count but are included in the waiting count for 1-2 PM, 2-3 PM, and 3-4 PM. To the best of our knowledge, no published studies have focused on predicting waiting count in US EDs using deep learning models. Since the literature on waiting count prediction is lacking, we summarize key studies on arrival count prediction, which most closely align with our research objective. For example, Harrou et al [6] developed deep learning models to predict hourly and daily ED arrival counts, comparing the performance of a variational autoencoder with 7 others: recurrent neural networks (RNNs), long short-term memory (LSTM), bidirectional long short-term memory (BiLSTM), convolutional LSTM, restricted Boltzmann machine, gated recurrent units, and convolutional neural networks (CNN). Tuominen et al [26] used advanced ML models, including probabilistic forecasting with autoregressive recurrent networks (DeepAR) [27], neural basis expansion analysis for interpretable time series forecasting (N-BEATS) [28], temporal fusion transformer, and light gradient boosting machine (LightGBM) [29], with multivariable inputs such as bed availability in catchment area hospitals, traffic data, and weather variables to predict daily ED occupancy. Similarly, Giunta et al [30] developed a multivariable predictive model based on the National Emergency Department Overcrowding Study score to predict sustained critical ED overcrowding lasting 8 or more hours, incorporating weather, patient flow, and bed occupancy variables. However, despite their advanced methodologies and multivariable approaches, none of these studies examined their predictions within the context of a proactive FCP.

Although these studies advance ED overcrowding prediction, each has limitations that reduce practical use in high-volume EDs. Many models have relied on a narrow set of inputs, such as patient arrivals or historical bed occupancy, without incorporating a broader range of operational, staffing, or environmental variables. In our study, we focus on forecasting the ED waiting count rather than the arrival count, as the arrival count only captures new patient entries and does not reflect ongoing congestion in the waiting area. In contrast, waiting count directly measures the number of patients present at any given time, providing a more accurate assessment of real-time crowding. Additionally, most studies have focused exclusively on daily metrics, failing to capture both hourly and daily fluctuations needed for real-time resource planning. To address these gaps, this study integrates hourly and daily predictions for more granular, actionable forecasting.

## Methods

### Ethical Considerations

This study was reviewed and approved by the Institutional Review Board (IRB) at the University of Alabama at Birmingham, with IRB# IRB-300011584.

### Research Framework

**Figure 1:**
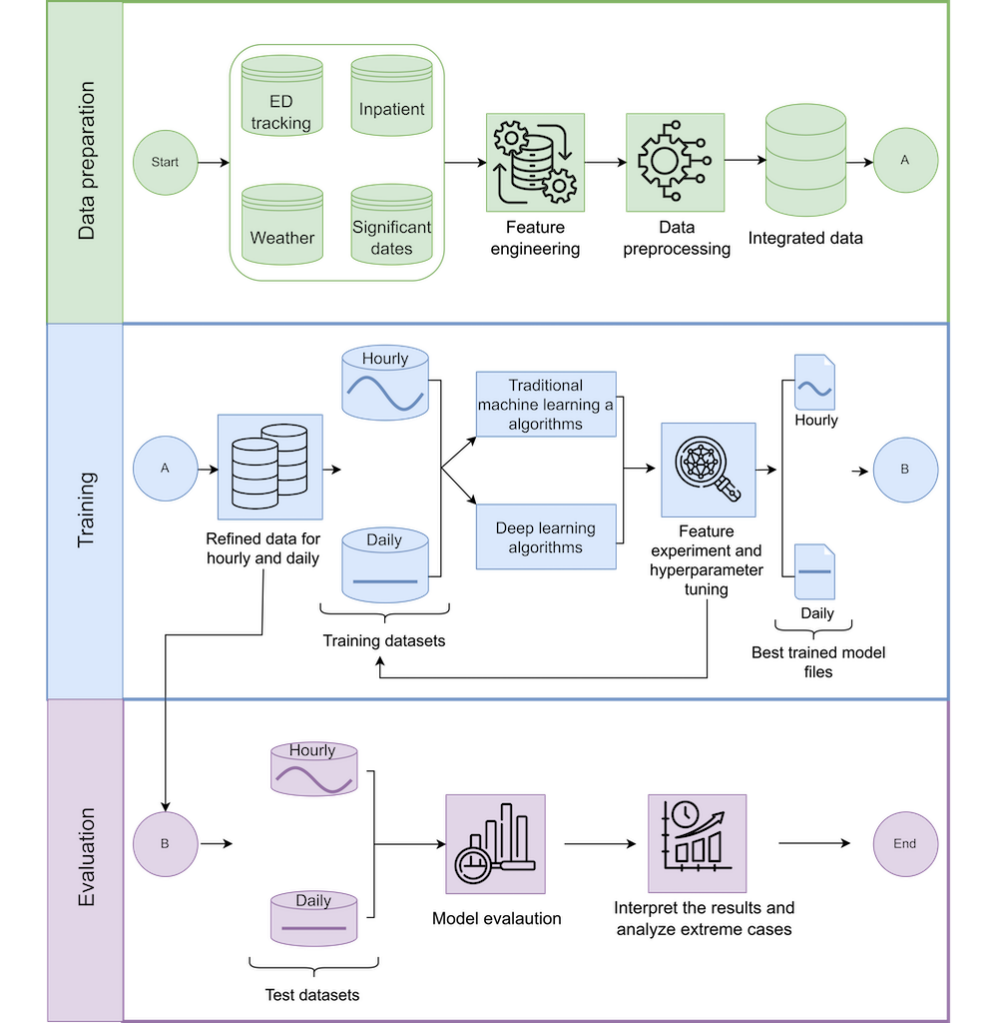
Proposed research framework. ED: emergency department.

Figure 1 shows our proposed framework, which has 3 main phases: data preparation, training, and evaluation. The framework includes 2 prediction approaches. The first provides hourly predictions, estimating the number of patients in the waiting room 6 hours ahead to support intraday resource management. The second provides daily predictions, estimating the average waiting count for the next 24 hours to help anticipate ED conditions for the following day. In this paper, patient counts in the waiting room are referred to as “waiting counts,” and their 24-hour average as “average waiting count.”

The data preparation phase involves processing data from 4 sources: the ED tracking system, inpatient records, weather information, and significant dates. After obtaining data from different sources, feature engineering and data preprocessing are applied separately to each source before creating the integrated final data. Following this, data preprocessing focuses on cleaning, scaling, and recategorizing certain categorical variables to prepare datasets for use in predictive modeling. After completing feature engineering and data preprocessing for each data source, all sources are integrated on an hourly basis into a single comprehensive dataset. The mathematical calculation of all features, including the target variable waiting count, along with all preprocessing and feature engineering steps, is described in detail in Multimedia Appendix 1 [31-56]. Table 1 includes descriptive statistics: numerical features show mean (SD), min, and max; categorical features indicate dataset percentages; and temporal features specify the time range used.

**Table 1:** Summary of the overcrowding dataset.

Features				Hourly		Daily
**Date range^a^**						
	Number of years		4			4
	Number of months		12			12
	Days of a month		1-31			1-31
	Days of a week		7			7
	Time (hours)		24			24
**Waiting count (target variable)**						
	Average (SD)		18.11 (9.77)			18.11 (4.51)
	Range		0-59			7-35
**Waiting count by ESI^b^ levels (%)**						
	ESI levels 1 and 2		26.38			26.38
	ESI level 3		58.34			58.34
	ESI levels 4 and 5		14.69			14.69
**Average waiting time (minutes)**						
	Average (SD)		90.98 (62.85)			90.98 (32.24)
	Range		0-425			9-170
**Average waiting time by ESI levels (minutes)**						
	**ESI levels 1** **and** **2**					
		Average (SD)	62.81 (69.98)		62.81 (31.69)	
		Range	0-538		5-162	
	**ESI level 3**					
		Average (SD)	106.9 (76.13)		106.9 (38.92)	
		Range	0-526		8-193	
	**ESI levels 4** **and** **5**					
		Average (SD)	56.03 (66.79)		56.03 (28.53)	
		Range	0-536		8-166	
**Treatment count**						
	Average (SD)		68.29 (23.69)			68.29 (22.64)
	Range		9-139			31-123
**Average treatment time (minutes)**						
	Average (SD)		52.93 (3.11)			52.93 (2.07)
	Range		28-60			46-57
**Boarding count**						
	Average (SD)		46.78 (29.60)			46.78 (29.30)
	Range		3-121			8-115
**Average boarding time (minutes)**						
	Average (SD)		54.06 (4.45)			54.06 (3.15)
	Range		12-60			42-58
Extreme case indicator (%)				3.1		28.77^c^
**Hospital census**						
	Average (SD)		794.23 (71.88)			794.23 (61.50)
	Range		584-1017			610-931
**Temperature (°F)**						
	Average (SD)		64.06 (15.36)			64.06 (13.60)
	Range		9-100			15-87
**Wind speed (m/s)**						
	Average (SD)		2.26 (1.97)			2.26 (1.22)
	Range		0-15			0-7
**Humidity (%)**						
	Average (SD)		73.38 (19.08)			73.38 (11.97)
	Range		15-100			40-97
**Weather status (%)**						
	Clouds		57.67			57.67
	Clear		22.89			22.89
	Rain		15.12			15.12
	Mist		2.55			2.55
	Thunderstorm		1.27			1.27
	Drizzle		0.16			0.16
	Fog		0.13			0.13
	Haze		0.12			0.12
	Snow		0.06			0.06
	Smoke		0.03			0.03
Number of football games				40		40
Number of federal holidays				34		34

^a^Data from January 1, 2020, to May 1, 2021, was excluded due to COVID-19.

^b^ESI: Emergency Severity Index.

^c^28.77% of rows have nonzero values, indicating days that experienced extreme patient volumes during at least some portion of the day.

During the training phase, the integrated data from the data preparation stage is used to generate 2 refined datasets: one for the hourly prediction approach and another for the daily prediction approach. Subsequently, 16 different dataset variations are created to be used by both approaches, each with distinct feature combinations, as detailed in Table S2 in Multimedia Appendix 2. A total of 11 ML algorithms, in Table S1 in Multimedia Appendix 1 [31-56], are used to train and evaluate models on both hourly and daily datasets to identify the best-performing models. These algorithms are categorized into four groups: (1) traditional machine learning (random forest [RF] [31] and extreme gradient boosting [XGBoost] [32]); (2) RNN-based models (LSTM) [33], BiLSTM [34], and sequence to sequence learning with neural networks [Seq2Seq] [35]; (3) CNN-based architectures (fully convolutional network plus [FCNPlus] [36], residual network plus [ResNetPlus] [37], XceptionTimePlus [38], and explainable convolutional neural network plus [XCMPlus] [39]); and (4) transformer-based models (time series transformer plus [TSTPlus] [40] and time series vision transformer plus [TSiTPlus] [41]). The architectures of all algorithms are explained in detail in Multimedia Appendix 1 [31-56]. Each combination of an algorithm and a dataset is considered a separate model in this study. To improve performance, different hyperparameter configurations are tested across the 16 dataset variations.

In the evaluation phase, performances of the trained models are evaluated. The best-performing models, selected for both hourly and daily prediction tasks, are tested using evaluation metrics detailed in the Model Evaluation section of Multimedia Appendix 1 [31-56]. The evaluation is primarily based on 4 different metrics: mean absolute error (MAE), mean squared error (MSE), root mean squared error (RMSE), and the coefficient of determination (*R*²). To further assess model effectiveness, the best-performing hourly prediction model is evaluated under extreme case scenarios, representing periods of exceptionally high patient volumes, with the results presented in Table 2. Additionally, the performance of the best hourly model is analyzed across the hour-of-day, capturing variations and trends in predictive accuracy at different times, as illustrated in Figure S3 in Multimedia Appendix 3.

**Table 2:** Performance of the time series vision transformer plus (TSiTPlus) in predicting extreme cases across different datasets.

Dataset	MAE^a^		
	Extreme (≥28) (mean + 1σ^b^)	Very extreme (≥38) (mean + 2σ)	Highly extreme (≥48) (mean + 3σ)
DS0	6.86	11.61	17.90
DS1	7.07	12.13	19.30
DS2	7.08	12.06	19.21
DS3	6.98	11.61	18.10
DS4	6.85	11.36	17.77
DS5	6.88	11.78	18.75
DS6	6.84	11.61	18.41
DS7	6.63	11.21	17.54
DS8	6.59	11.07	17.14
DS9	6.57	11.13	17.35
DS10	6.58	11.11	17.15
DS11	6.31	10.69	16.76
DS12	6.31	10.70	16.82
DS13	6.18	10.44	16.78
DS14	6.16^c^	10.16^c^	15.59^c^
DS15	6.31	10.45	16.16

^a^MAE: mean absolute error.

^b^σ: SD.

^c^Lowest MAE.

### Data Sources

The data sources in this study cover the period from January 2019 to July 2023 and are categorized into hospital data, including ED tracking system and inpatient records, and external data, consisting of weather information and significant dates for football games and federal holidays.

The ED tracking data provides detailed records of patient arrivals and departures within the ED waiting and treatment rooms of our partner hospital. This data also includes ESI levels indicating acuity, patient classifications, room types, event status (eg, complete, request, and cancel), and other ED-related information. Both waiting room and treatment room identifiers are recorded, enabling comprehensive tracking of patient movements from arrival to discharge or inpatient admission through unique patient and visit identifiers. The data contains 161,477 unique patients and 308,196 unique visits.

The inpatient data contains time-stamped records of patient admissions to and discharges from inpatient units. These records enable the hourly calculation of the hospital-wide patient census feature, as shown in Table 1. The dataset comprises 293,716 unique inpatient visits for 180,589 unique patients, representing hospital-wide admissions encompassing the study period.

The external data includes weather data and significant events, which are federal holidays and local football game event data. The weather data provides hourly weather information collected from a nearby weather station located close to the partner hospital. This data is sourced from the historical data archive of the OpenWeatherMap History Bulk [57] and includes numerical variables such as temperature, humidity, and wind speed, as well as a categorical variable indicating categories of clear skies, clouds, rain, mist, thunderstorm, snow, drizzle, haze, fog, and smoke. The significant dates consist of 2 external datasets: federal holidays and football game dates for a major team near the hospital. On average, there are 13 football games and 10 federal holidays each year. Federal holidays were obtained from the United States Office of Personnel Management Government website [58], and football game dates were sourced from the team’s official website [59].

### Problem Modeling

To predict waiting counts in the ED waiting room at a time point *h* steps into the future (eg, predicting 6 hours from now, or any other chosen future time point), we model this problem as a time series ML problem. We set up the problem as a direct single-step forecast targeting *y*_t+h_ from the current time *t*. Let:

- *y*_t_ represent the waiting count at the current time *t*.
- *h* be the prediction horizon (ie, the number of time steps ahead for the forecast (eg, *h*=6 for 6 hours ahead).
- *k* denote the number of past observations (lags) included in the model.
- (*l*_1_, *l*_2_,…, *l*_N_) represent variables that do not require lagging (eg, weather conditions, holidays).
- *x*_t_, *x*_t–1_,…, *x*_t–k+1_ represent any feature other than the waiting count that has a lag.
- *g*(.) be the function learned by the ML model, trained using historical data.

The model can be expressed as follows:



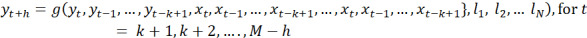



Where:

- *y*_t+h_ is the predicted waiting count at time *t*+*h*.
- *y_t_*, *y*_*t*–1_,…, *y*_*t–k*+1_are waiting count lag features to capture temporal dependencies.
- *M* is the total number of observations and the training dataset size is *M–k–h*+1.

### Model Design

Model design involves selecting the relevant feature combinations for prediction. With a total of 34 main features, excluding lag and rolling mean features, determining the optimal feature set is an essential step in improving model performance. Therefore, numerous experiments were conducted initially to refine feature combinations, scaling methods, COVID-19 date exclusion ranges, weather categorizations, and hyperparameter ranges. Based on the outcomes of these experiments, a total of 16 datasets were generated, as shown in Table S2 in Multimedia Appendix 2, each containing different combinations of features. These combinations were selected through a manual but systematic approach guided by domain knowledge and performance feedback. Specifically, we varied key dimensions such as lag lengths, rolling window sizes, weather encodings, temporal coverage, and the inclusion of engineered variables like the extreme case indicator. Each dataset was crafted to test how individual or grouped feature categories (eg, ED tracking, inpatient census, weather, significant dates) influence model generalization and forecasting accuracy. To evaluate these combinations, 11 different algorithms, as detailed in Table S1 in Multimedia Appendix 1 [31-56], were applied to train models on each dataset. The primary objective was to identify the most effective combination of features and algorithms for achieving optimal performance. For both hourly and daily prediction models, a total of 8800 experiments (11 algorithms × 16 datasets × 50 trials) were conducted separately to evaluate these combinations and determine the best-performing model and feature set.

### Hourly and Daily Time Horizons

To address the varying forecasting needs in ED, this study uses 2 types of predictive models: hourly and daily, as illustrated in Figure S4 in Multimedia Appendix 3. These models were developed to handle different prediction horizons, allowing for more comprehensive management of patient flow in the ED.

The hourly model predicts the waiting count 6 hours ahead. As shown by the red arcs in Figure S4 in Multimedia Appendix 3, this model provides flexibility by not being restricted to specific times of the day. Instead, it generates forecasts for 6 hours into the future whenever it runs, enabling it to be applied at any time when updated predictions are needed.

The daily model predicts the average waiting count for the next 24-hour period, running each day at 5 PM and using data from the previous 24 hours (5 PM the previous day to 5 PM today). At each run, it provides a single estimate for the upcoming 24-hour window (5 PM today to 5 PM tomorrow), offering insights that aid in planning for the next day. Figure S4 in Multimedia Appendix 3 shows the daily model’s prediction timeline, represented by the orange elements, where forecasts are generated once per day to estimate the average waiting count in the waiting room for the next 24-hour period.

The selection of the 2 prediction horizons—6 hours ahead and daily—was informed by consultations with hospital operational leadership and the clinical advisory board to align with real-world decision-making workflows. The 6-hour-ahead forecast supports intrashift planning by enabling proactive resource allocation for anticipated short-term crowding. The daily forecast facilitates long-range planning, including staffing, bed coordination, and potential FCP activation based on projected 24-hour patient volumes. Together, these models address both immediate and next-day operational needs.

## Results

### Overview

We present and analyze the evaluation results of 11 ML algorithms (listed in Table S1 in Multimedia Appendix 1 [31-56]) applied to 16 different datasets (described in Table S2 in Multimedia Appendix 2). The results cover 2 separate prediction approaches: one focuses on forecasting the waiting count 6 hours ahead, and the other estimates the average waiting count over the next 24-hour period.

### Performances of Hourly Prediction Models

**Figure 2:**
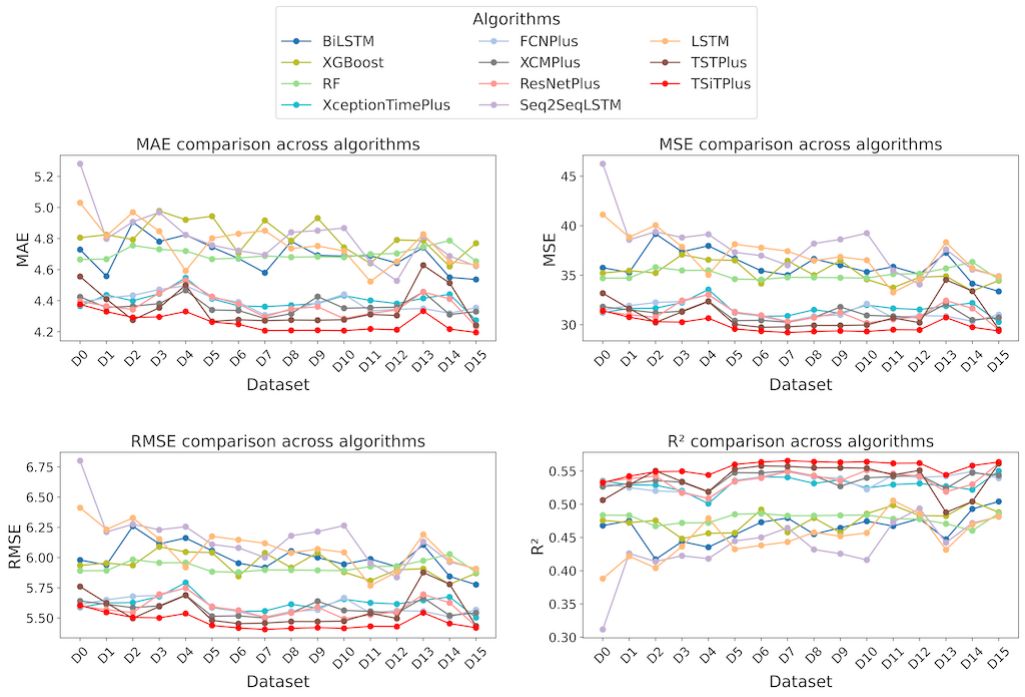
Comparison of hourly model performances across 16 datasets and 11 algorithms based on different evaluation metrics. BiLSTM: bidirectional long short-term memory; FCNPlus: fully convolutional network plus; LSTM: long short-term memory; MAE: mean absolute error; MSE: mean squared error; ResNetPlus: residual network plus; RF: random forest; RMSE: root mean squared error; Seq2Seq: sequence to sequence learning with neural networks; TSiTPlus: time series vision transformer plus; TSTPlus: time series transformer plus; XCMPlus: explainable convolutional neural network plus.

The target variable waiting count, which represents the number of patients in the waiting room during a given hourly interval, has a mean value of 18.11 and a SD of 9.77, as shown in Table 1. Figure 2 presents the performance analysis of ML models in predicting waiting counts 6 hours ahead across 16 different datasets (DS0 to DS15). In Figure 2, the x-axis in all subplots represents the datasets, while the y-axis corresponds to each evaluation metric. Among the evaluated models, the TSiTPlus algorithm demonstrated the best overall performance, achieving an MAE of 4.19, an MSE of 29.36, an RMSE of 5.42, and an *R*² of 0.56 on dataset DS15.

As shown in Figure 2, traditional ML algorithms, RF and XGB, displayed similar performance patterns, with RF achieving its best results on dataset DS15 and XGB on dataset DS14. Specifically, RF’s performance on DS15 yielded an MAE of 4.65, an MSE of 34.56, an RMSE of 5.87, and an *R*² of 0.48, using 100 estimators, a maximum depth of 30, and 4 samples per leaf, with bootstrapping enabled. Across all datasets, RF’s MAE varied between 4.65, its best performance, and 4.78, its worst performance. On the other hand, XGB achieved slightly better values on dataset DS14, with an MAE of 4.62, an MSE of 33.40, an RMSE of 5.78, and an *R*² of 0.50, with a maximum depth of 15, a learning rate of 0.02, a subsample ratio of 0.8, and a column sampling rate of 0.3. These results highlight the limitations of traditional algorithms, which treat each observation independently and lack the ability to capture the long-term dependencies and complex sequential patterns inherent in time-series data.

RNN-based models, including Seq2Seq, LSTM, and BiLSTM, delivered better results than traditional ML algorithms for predicting waiting counts 6 hours ahead. Among these, Seq2Seq achieved its best performance on dataset DS12, with an MAE of 4.52, an MSE of 34.06, an RMSE of 5.83, and an *R*² of 0.49. This result was obtained using a batch size of 32, a learning rate of 0.01, a weight decay of 0.2, a dropout rate of 0.1, and Adam as the optimization function. The Seq2Seq model’s performance, based on MAE, ranged from its worst result of 5.28 on dataset DS0 to its best result of 4.52 on dataset DS12. LSTM demonstrated consistent results across datasets, delivering its best performance on dataset DS11, with an MAE of 4.52, an MSE of 33.26, an RMSE of 5.76, and an *R*² score of 0.505. This result was achieved using a batch size of 32, a learning rate of 0.01, a dropout rate of 0.2, a weight decay of 0.1, and stochastic gradient descent as the optimization algorithm [60]. The LSTM model’s performance, based on MAE, ranged from its worst result of 5.03 on dataset DS0 to its best result of 4.52 on dataset DS11. BiLSTM, which leverages its bidirectional architecture, achieved its best results on dataset DS15, with an MAE of 4.54, an MSE of 34.67, an RMSE of 5.89, and an *R*² score of 0.48. However, it encountered higher errors on datasets such as DS2, where the MAE was 4.91.

The CNN-based algorithms, including ResNetPlus, XceptionTimePlus, FCNPlus, and XCMPlus, demonstrated better performance than both traditional ML and RNN-based algorithms across multiple datasets in this study. Among these, ResNetPlus achieved the best overall performance, particularly on dataset DS15, with an MAE of 4.23, an MSE of 29.93, an RMSE of 5.47, and an *R*² of 0.555. XceptionTimePlus achieved its best results on dataset DS15 as well, with an MAE of 4.274, an MSE of 30.279, an RMSE of 5.503, and an *R*² of 0.55. FCNPlus performed best on dataset DS7, with an MAE of 4.3081, an MSE of 30.3208, an RMSE of 5.5064, and an *R*² of 0.5491. Finally, XCMPlus delivered its best results on dataset DS7, with an MAE of 4.285, an MSE of 30.254, an RMSE of 5.5, and an *R*² of 0.55. These results suggest that CNN-based models generally provided more accurate predictions than traditional and RNN-based algorithms.

The transformer-based models, TSiTPlus and TSTPlus, delivered better results compared with other algorithm categories in this study, with TSiTPlus being the best-performing model based on MAE. TSiTPlus achieved its best performance on dataset DS15, with an MAE of 4.19, an MSE of 29.36, an RMSE of 5.41, and an *R*² of 0.56. In comparison, TSTPlus showed good performance but had slightly higher errors. Its best results were on dataset DS15, with an MAE of 4.24, an MSE of 29.52, an RMSE of 5.43, and an *R*² of 0.561. DS15 incorporated a range of features, including waiting count lags, rolling averages, patient flow indicators, weather conditions, hospital census data, and significant event markers. Across other datasets, TSTPlus achieved MAE values such as 4.27 on DS7 and 4.27 on DS8. The performance of TSiTPlus indicates that it was the most accurate model in this study for predicting waiting counts. To examine the impact of including the COVID-19 period, TSiTPlus was evaluated on DS15, the best-performing model and dataset combination. The results indicated noticeably higher prediction errors (MAE: 4.82 vs 4.19; MSE: 40.22 vs 29.32), suggesting that patient flow anomalies during the pandemic adversely affected model performance.

TSiTPlus demonstrated superior performance over all other models in hourly predictions. Specifically, it achieved a 9.8% reduction in mean MAE compared with RF (MAE=4.65), a 7.3% reduction compared with the best-performing RNN-based model, Seq2Seq (MAE=4.52), and a 0.9% improvement over the best CNN-based model, ResNetPlus (MAE=4.23). Although the performance gap between TSiTPlus (MAE=4.19) and the best traditional ML model, XGBoost (MAE=4.62), may initially appear modest, its operational significance becomes clear when scaled to the continuous nature of ED operations. Given that predictions are generated hourly—24 times each day—this 0.43 improvement in MAE results in approximately 10.3 (0.43 × 24 hours), more accurate waiting count forecasts per day. When extended over an entire year, this translates to 3767 waiting counts (10.3 × 365 days), more accurate predictions, each representing a more informed decision point for hospital staff.

To estimate uncertainty around our model’s point estimates, we applied a bootstrap resampling approach [61], which repeatedly samples from the original test data to approximate the sampling distribution of evaluation metrics. Specifically, we used block bootstrapping with our best-performing model, TSiTPlus, on DS15. A block size of 24 was chosen to match the 24-hour lag window, preserving local temporal dependencies inherent in hourly hospital data. For each iteration, blocks of 24 consecutive hours were resampled with replacement from the test dataset to form a new evaluation set, and performance metrics were computed. This method yielded robust IQRs for each metric. As shown in Table 3, MAE reaches 4.19 with an IQR of 4.16-4.25, varying by only 0.09 across samples—indicating highly consistent model performance. MSE reaches 29.36 (IQR 28.65-30.20), RMSE reaches 5.42 (IQR 5.35-5.49), and *R*² reaches 0.56 (IQR 0.55-0.57); all similarly demonstrate narrow IQRs, further supporting the stability and reliability of the model’s predictions. These narrow IQRs suggest that the model is not only accurate but also reliable under different test-time conditions.

**Table 3:** Bootstrap-based uncertainty estimates for model performance metrics using Time Series Vision Transformer Plus (TSiTPlus) on dataset DS15.

Model and metric			Median (IQR)
**TSiTPlus**			
	MAE^a^	4.19 (4.16-4.25)	
	MSE^b^	29.36 (28.65-30.20)	
	RMSE^c^	5.42 (5.35-5.49)	
	*R* ^2^	0.56 (0.55-0.57)	

^a^MAE: mean absolute error.

^b^MSE: mean squared error.

^c^RMSE: root mean squared error.

The strong performance of TSiTPlus in this study can be attributed to its ability to capture complex patterns in multivariate time series data. As a transformer-based model, it is well-suited for recognizing relationships and trends over time. TSiTPlus divides the time series into smaller segments, treating them as input tokens—similar to words in a sentence—which allows the model to capture both short- and long-term patterns. This design enables it to learn from multiple timeframes, making it particularly effective for datasets with complex temporal dynamics.

### Analysis of Extreme Case and Hour-of-Day

Table 2 presents the performance of TSiTPlus, the best-performing algorithm, in predicting extreme cases across various datasets. For “Extreme Cases” (mean + 1σ), MAE ranged from 6.16 on DS14 to 7.08 on DS2. In “Very Extreme Cases” (mean + 2σ), MAE ranged from 10.16 on DS14 to 12.13 on DS1. For “Highly Extreme Cases” (mean + 3σ), TSiTPlus achieved its best result on DS14 with an MAE of 15.59. When averaging errors across all 3 categories, DS14 performed best, followed by DS15, demonstrating strong predictive capabilities under high patient volume. DS14’s superior performance is likely due to its 48-hour lag features, which incorporate data from the past 2 days (Table S2 in Multimedia Appendix 2).

Our best-performing model not only predicts typical waiting counts accurately but also performs well under extreme crowding. Compared with traditional models, such as RF (MAE: 14.0 for Very Extreme, 20.0 for Highly Extreme) and XGBoost (MAE: 13.9 for Very Extreme, 19.6 for Highly Extreme), TSiTPlus consistently shows lower error rates during critical situations. This reduction in prediction error during peak periods allows for earlier, more accurate interventions, improving ED responsiveness and resource coordination. When evaluating the cumulative effect of prediction performance over time, the difference becomes even more meaningful. For example, in very extreme scenarios, TSiTPlus achieves an MAE of 10.16 compared with XGBoost’s 13.9—an improvement of 3.74. This translates to approximately 90 (3.74 × 24 hours) more accurate waiting count predictions per day. When applied year-round, this provides a clear operational advantage for anticipating and managing ED overcrowding.

Figure S3 in Multimedia Appendix 3 shows the TSiTPlus model’s performance by hour of day. It performs best between 10 PM and 6 AM, with MAE under 4 and RMSE between 3 and 5.25, reflecting more stable patient volumes. Error metrics increase between 6 PM and 8 PM, where MAE nears 5 and RMSE exceeds 6, likely due to higher variability in waiting counts during this time.

### Performance of Daily Prediction Models

**Figure 3:**
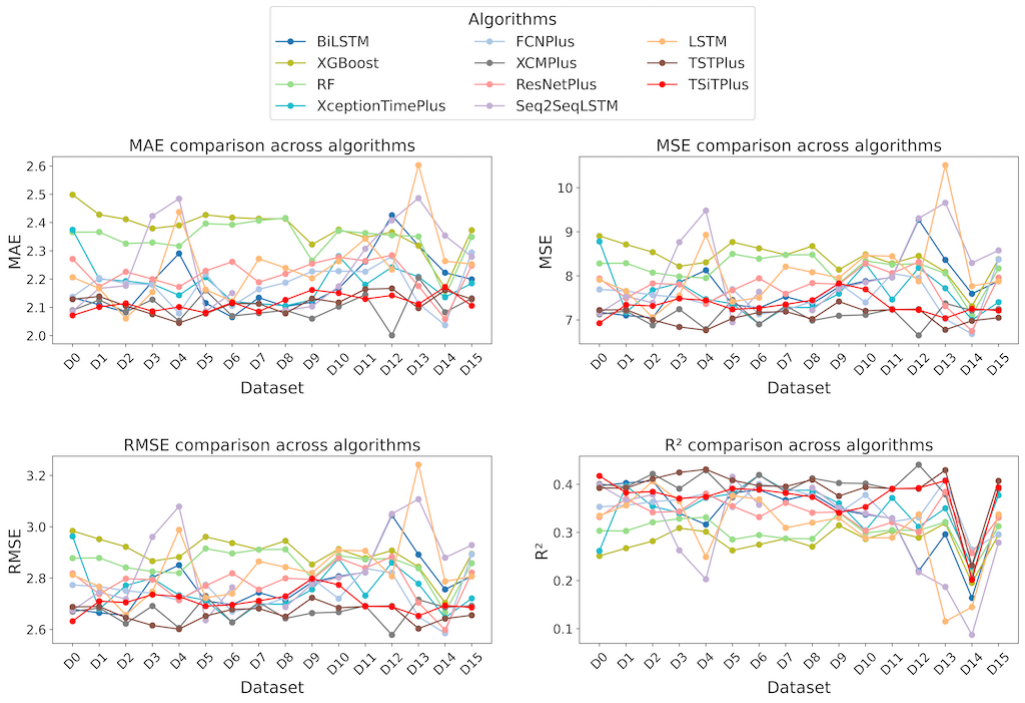
Comparison of daily model performance across 16 datasets based on different evaluation metrics. BiLSTM: bidirectional long short-term memory; FCNPlus: fully convolutional network plus; LSTM: long short-term memory; MAE: mean absolute error; MSE: mean squared error; ResNetPlus: residual network plus; RF: random forest; RMSE: root mean squared error; Seq2Seq: sequence to sequence learning with neural networks; TSiTPlus: time series vision transformer plus; TSTPlus: time series transformer plus; XCMPlus: explainable convolutional neural network plus.

As shown in Figure S4 in Multimedia Appendix 3, daily prediction is conducted at 5 PM each day to estimate the next day’s average waiting count. The target variable has a mean of 18.11 and an SD of 4.51 (Table 1). Daily prediction errors are lower than hourly due to smoother target values and a smaller dataset. Using the same 11 ML algorithms (Table S1 in Multimedia Appendix 1 [31-56]) across 16 datasets (Multimedia Appendix 2), the best performance was achieved by XCMPlus with an MAE of 2.00, MSE of 6.64, RMSE of 2.57, and *R*² of 0.44, as shown in Figure 3.

Traditional ML algorithms, RF and XGBoost, demonstrated similar performance across all 16 dataset configurations for daily predictions, as illustrated in Figure 3. Both algorithms achieved their best results on dataset DS14, with RF slightly outperforming XGBoost. Specifically, RF recorded the lowest MAE of 2.13, MSE of 7.10, RMSE of 2.66, and an *R*² of 0.217, while XGBoost followed closely with an MAE of 2.16, MSE of 7.31, RMSE of 2.70, and an *R*² of 0.195. The performance gap between the best and worst configurations for both algorithms, with RF ranging from 2.13 to 2.41 MAE and XGBoost from 2.16 to 2.49 MAE, indicates consistent behavior across different feature combinations. RF achieved its optimal performance with 200 estimators, a maximum depth of 40, a minimum sample split of 10, and 8 samples per leaf with bootstrapping enabled. Similarly, XGBoost’s best configuration used a dart booster with a maximum depth of 12, learning rate of 0.02, subsample ratio of 0.8, column sampling rate of 0.4, and weighted sampling with a rate drop of 0.2.

The analysis of the RNN-based models, including LSTM, BiLSTM, and Seq2Seq, shows varying performance across datasets. The LSTM model performs consistently, with MAE values between 2.06 and 2.60 and RMSE between 2.65 and 3.24, achieving the lowest MAE on dataset DS2 and the highest on dataset DS13. BiLSTM performs slightly better, with MAE ranging from 2.06 to 2.42 and RMSE from 2.65 to 3.07, showing the best performance on dataset DS6 and the highest error on dataset DS12. Seq2Seq provides competitive results, with MAE between 2.08 and 2.48 and RMSE from 2.63 to 3.10, performing best on dataset DS5 and worst on dataset DS13. The *R*² values indicate that all models exhibit moderate predictive power, with the highest values around 0.41 and the lowest around 0.11. Among the 3 models, BiLSTM offers the best balance between low MAE and stable *R*² values, while Seq2Seq shows more consistent performance across datasets.

The analysis of CNN-based models, including FCNPlus, ResNetPlus, XceptionTimePlus, and XCMPlus, shows varying performance across datasets. FCNPlus has MAE values from 2.03 to 2.44 and RMSE between 2.58 and 3.01, performing best on dataset DS14 and worst on dataset DS11, with *R*² values ranging from 0.26 to 0.23. ResNetPlus shows slightly better performance, with MAE values between 2.05 and 2.40, RMSE from 2.59 to 3.02, and *R*² values between 0.25 and 0.22. XceptionTimePlus provides competitive performance, with MAE between 2.10 and 2.33 and RMSE from 2.71 to 2.88, achieving its best results on dataset DS8 and the highest error on dataset DS11. XCMPlus outperforms the other models, with the lowest MAE (2.00 to 2.28) and RMSE (2.57 to 2.92), showing the best performance on dataset DS12 and the highest error on dataset DS0. DS12 incorporated waiting count by ESI levels, treatment and boarding counts, and weather conditions, with rolling means and lag features enhancing trend detection. These features contributed to its strong predictive performance, especially for XCMPlus. It also has the highest *R*² values, ranging from 0.44 to 0.28, indicating better predictive accuracy. Overall, XCMPlus demonstrates the best performance among all algorithms, not just CNN-based models, achieving the lowest MAE and highest *R*² values, making it the most effective model for predicting daily waiting counts across datasets.

The analysis of the transformer-based models, TSiTPlus and TSTPlus, highlights differences in their performance across datasets. TSiTPlus demonstrates MAE values ranging from 2.08 to 2.17 and RMSE values between 2.63 and 2.80, with the best performance observed on dataset DS5 and the highest error on dataset DS14. The *R*² values for TSiTPlus vary from 0.41 to 0.20, indicating moderate predictive accuracy. TSTPlus achieves slightly lower MAE values compared with TSiTPlus, ranging from 2.04 to 2.18, and RMSE values between 2.57 and 2.70. The best results

are observed for dataset DS4, while the highest error occurs on dataset DS11. The *R*² values range from 0.42 to 0.27, showing slightly better predictive power compared with TSiTPlus. Overall, TSTPlus shows slightly better performance than TSiTPlus in terms of lower MAE and higher *R*² values across datasets, making it the more effective transformer-based model for predicting waiting counts.

## Discussion

### Principal Findings

This study developed time series ML models to forecast ED waiting room counts using real-world operational and contextual data. TSiTPlus achieved the best 6-hour-ahead prediction performance (MAE: 4.19, RMSE: 5.42, *R*²: 0.56 on DS15), while XCMPlus performed best for daily forecasts (MAE: 2.00, RMSE: 2.57, *R*²: 0.44 on DS12). MAE differences across datasets for TSiTPlus and XCMPlus were minimal in hourly predictions; however, the extreme case analysis revealed greater variation, underscoring the importance of feature engineering. For the hourly prediction models, comparing datasets highlights the incremental impact of added features. For example, the MAE for TSiTPlus decreased from 4.29 in DS2 to 4.26 in DS5 with the inclusion of weather status and ESI-based waiting counts. Incorporating significant dates such as football games and federal holidays further improved performance, reducing the MAE to 4.24 in DS6. Additional operational features—like treatment count, boarding count, and related time metrics—lowered the MAE to 4.21 in DS8. Further adjustments, such as different lagged features and rolling means, resulted in only minor MAE changes across DS8 to DS14. Ultimately, the best performance was achieved with DS15, where TSiTPlus reached an MAE of 4.19 after integrating all available features. For the daily prediction models, the best accuracy was achieved using DS14, which shares the same feature combination as the best hourly dataset, DS15, except that DS14 does not include temperature, humidity, wind speed, or football match variables. This suggests these variables do not significantly impact long-term average waiting count predictions. These results demonstrate the potential of deep learning–based time series models to support proactive FCP implementation through accurate, time-sensitive predictions. Additionally, we evaluated the impact of including COVID-19 period data for TSiTPlus on DS15. Contrary to some findings in the literature [62], which reported improved results for arrival count prediction when COVID-19 data was included, our results showed that adding the pandemic period to our training data negatively affected model performance for waiting count prediction (MAE increased from 4.19 to 4.82; MSE increased from 29.32 to 40.22). This suggests that the atypical patient flow patterns during the COVID-19 pandemic introduced additional variability, making it harder for the model to accurately capture or reflect standard ED operations.

### Managerial Impact

EDs often face overcrowding due to challenges such as limited resources [6], high patient volumes [7], and inefficient patient flow management [8]. This represents an initiative to transform reactive FCP to be proactive by developing predictive models that forecast a key PFM, which is ED waiting counts. The prediction is done at two distinct time scales—6 hours ahead and 24 hours in advance—allowing for both hourly operational adjustments and daily planning. The selection of these prediction horizons was based on input from the hospital’s PFCT and the project advisory board. The 6-hour window was identified as a critical lead time for initiating immediate operational responses such as surge staffing, bed reassignments, and overflow room activation. In contrast, the 24-hour forecast was designed to inform daily planning activities, including staff scheduling, diversion decisions, and intensive care unit (ICU) bed coordination. Predictions with longer windows can provide a greater margin for operational adjustments, making it easier for hospitals to mobilize resources and take proactive action when surges are anticipated. However, evidence is still lacking as to which prediction window offers the most sustainable and actionable foundation for managing real-world ED surges, since practical constraints and response capabilities can vary significantly between institutions.

The hourly prediction model, which predicts the total waiting count in the next 6 hours, enables real-time resource allocation by giving hospital managers enough time (eg, 6 hours) to proactively implement FCP interventions and resources that help mitigate ED crowding, such as adjusting staffing, mobilizing overflow spaces, and ensuring the availability of critical equipment before surges occur. For example, if the model predicts a peak in patient volume during the evening shift, managers can activate a surge plan, offering voluntary short-term shifts to nurses or physicians who opt in to alleviate crowding. Additionally, they can offer overtime to extend staff hours, call in additional clinical staff, or reassign staff to the ED ahead of anticipated crowding. These proactive measures help prevent bottlenecks, ensuring adequate coverage during peak times while minimizing unnecessary overtime costs. This capability minimizes understaffing during peak times while reducing unnecessary overtime costs. Additionally, FCP interventions—such as coordinating with inpatient units to expedite bed turnover or opening additional inpatient surge capacity, such as unstaffed beds or hallways, to absorb some of the ED boarding patients and relieve crowding—can be activated before crowding worsens, improving compliance with key performance indicators such as Centers for Medicare & Medicaid Services door-to-provider times and reducing “left without being seen” rates, which impact hospital revenue and reputation. These FCP interventions are not reserved for rare or exceptional circumstances but are routinely needed in everyday ED operations due to the persistent and dynamic nature of crowding.

The daily prediction model, which estimates the average patient waiting count over the next 24 hours (ie, from 5 PM on the current day to 5 PM the following day), supports broader decision-making processes. By forecasting average daily patient volumes, managers can optimize next-day staffing schedules and implement strategic interventions such as placing the hospital on diversion to temporarily halt nonessential incoming transfers, thereby preserving ED and inpatient capacity for critical cases. These predictions also enhance cross-departmental collaboration by facilitating proactive ICU bed reservations for anticipated ED admissions or diverting nonurgent cases to alternative care settings, such as urgent care clinics, to alleviate ED congestion. Over time, insights from daily predictions inform long-term capacity planning, such as adjusting seasonal budgets for flu surges or expanding ED infrastructure to accommodate growth trends.

The ability to act in advance is supported by the model’s predictive accuracy, particularly under extreme crowding scenarios. This is critical because the most impactful decisions—such as activating surge capacity, reallocating staff, or adjusting admissions—are typically required when the ED is under the most pressure. Having reliable forecasts during these peak periods ensures that hospital managers can make timely, confident decisions that reduce overcrowding and maintain the quality of patient care.

The practical implications of this study go beyond forecasting accuracy, offering direct support for real-world hospital operations. By leveraging both internal operational features—such as patient flow metrics, boarding counts, treatment activity, and hospital census—and external contextual features—including weather conditions, federal holidays, and major local events—the models provide actionable insights tailored to each facility’s environment. For example, if the 6-hour-ahead model forecasts a surge in ED waiting counts during an upcoming evening shift, hospital managers can proactively deploy surge nurses, call in backup physicians, prepare overflow treatment areas, or expedite inpatient bed turnover hours in advance. In parallel, daily forecasts indicating above-average volumes can be used during morning planning meetings to revise staffing levels, reserve ICU beds, or activate diversion protocols for nonurgent cases. In cases of anticipated extreme crowding, hospitals may also delay elective admissions, accelerate discharge rounds, or mobilize unstaffed bed capacity. Embedding these forecasts into hospital dashboards or integrating them with electronic health record systems enables timely alerts and streamlined workflows. By incorporating both operational and external drivers of demand, the system supports proactive, risk-informed decision-making that improves patient throughput, reduces waiting times, and enhances overall ED efficiency.

### Limitations and Future Work

A key limitation of this study is that we did not evaluate how the predictions directly support proactive FCP implementation. While our models can anticipate patient surges, their real-world impact on operational decision-making remains untested. In a future study, we will address this by using discrete event simulation to compare reactive versus proactive FCP strategies, assessing how prediction-driven interventions influence resource allocation, patient flow, and ED performance metrics.

Short-notice staffing adjustments can be challenging in ED settings, and a 6-hour notice may be insufficient for several reasons. Many clinical staff may be unable to respond to requests for additional coverage with such limited advance notice, particularly during nights or weekends. Longer-term forecasting horizons—such as 24-hour, weekly, or monthly predictions—may provide a more practical foundation for surge planning. While many studies have adopted daily or multiday horizons [21,22,26,63,64], the literature does not provide clear evidence about which prediction window is most effective in real-world practice. Given these constraints, future work will include a systematic evaluation of prediction windows across both short and long horizons to identify the most practical and operationally relevant timeframes for ED staffing.

Refinement of model adaptability and incorporation of more external data sources could further enhance predictive performance and real-world applicability. Another important consideration is the potential for data drift, including both covariate drift [65] and concept drift [66] over time. As clinical workflows, hospital policies, and external conditions evolve, model performance may degrade. To mitigate this risk, future deployments should incorporate drift detection mechanisms and model monitoring systems to ensure consistent forecast accuracy and trigger retraining when necessary.

The proposed system is designed for real-time operation, and a key direction for future work is its pilot deployment in a live clinical environment. This will be accompanied by a simulation-based evaluation to assess how the system integrates into operational workflows and influences decision-making. This study represents one component of a larger project, specifically focusing on the development of a predictive model for one of the PFMs—the waiting count. The broader project includes additional modules to predict other PFMs, such as boarding count and average waiting time, which will collectively support a comprehensive decision-support system for ED operations. Building on this, our future work will extend beyond standard ML workflows by implementing a real-time deployment architecture tailored for hospital operations. The planned deployment pipeline will run within the partner hospital’s internal supercomputing infrastructure, ingesting hourly data streams from the ED tracking system, inpatient units, weather application programming interfaces, and event calendars. We will consider technologies such as columnar storage formats (eg, Apache Parquet [67]) and embedded analytical engines (eg, DuckDB [68]) to enable low-latency access to recent data. A containerized preprocessing pipeline using Docker [69] is envisioned to ensure consistent and reproducible real-time data cleaning and feature engineering. Model lifecycle management, including data versioning and experiment tracking, will be managed by tools such as MLflow [70], enabling continuous preprocessing, inference, and updates as new data arrives. Predictions will ultimately feed into a decision support module to provide early warnings for crowding, with results visualized on an internal dashboard to enhance operational planning. Additionally, future efforts will address key implementation steps such as ensuring data privacy, developing user-facing interfaces aligned with clinical workflows, and conducting usability testing. The goal is to build a fully automated infrastructure for continuous monitoring, model retraining, and maintenance, supporting long-term reliability and clinical utility.

### Conclusions

The results of this study show that advanced ML approaches are effective in predicting ED waiting counts based on real-world data from our collaborating hospital. By integrating multiple datasets—ED tracking, inpatient data, weather, and significant dates—the research developed hourly and daily prediction models. The hourly model provides real-time, 6-hour-ahead forecasts, enhancing decision-making and resource allocation. The daily models offer insights into patient volumes at daily averages, aiding in operational planning.

A comprehensive evaluation was conducted by testing 11 different ML algorithms on 16 distinct datasets, each generated through careful feature engineering and hyperparameter optimization to explore optimal feature combinations. The results revealed that for the hourly prediction approach (6-hour-ahead prediction), the TSiTPlus algorithm consistently delivered the best performance, with DS15 emerging as the most effective dataset. DS15 incorporated a diverse range of features, including waiting count lags, rolling averages, patient flow indicators, weather conditions, hospital census data, and significant event markers. This combination enabled the model to achieve an MAE of 4.19, MSE of 29.36, RMSE of 5.42, and *R*² of 0.56, outperforming all other datasets. For the daily prediction approach (predicting the next 24-hour average waiting count), the best performance was achieved by the XCMPlus model using DS12, with an MAE of 2.00, MSE of 6.64, RMSE of 2.57, and an *R*² of 0.44. Through the creation and analysis of multiple feature sets, we identified the most effective feature combinations for improving prediction accuracy, demonstrating the critical role of feature engineering in model optimization.

This study also incorporated detailed extreme case and hour-of-the-day analyses to better understand prediction performance under various conditions. Extreme case analysis specifically evaluated how well the model predicts waiting counts during periods of severe overcrowding, defined as values exceeding the mean by 1, 2, or 3 SDs. The hour-of-the-day analysis further highlighted performance variations throughout the day, providing actionable insights into periods of higher prediction uncertainty and variability. This research paper represents one component of a larger decision support system initiative; in the future, hourly and daily prediction models—alongside additional PFMs such as boarding count and treatment count—will be integrated and operated together as part of a comprehensive, real-world decision-support system for ED management. Such a system can improve resource allocation, optimize staffing, and enhance overall operational efficiency. By leveraging the hourly model’s real-time forecasts and the daily model’s planning insights, ED administrators can make data-driven decisions to better handle patient flow, reduce overcrowding, and ensure timely care delivery.

## Appendix

Multimedia Appendix 1 Summary of methods covering feature engineering, data integration, model training, and evaluation.

Multimedia Appendix 2 Features and their combinations for dataset variants.

Multimedia Appendix 3 Distribution of hourly waiting counts with extreme thresholds, hourly mean and standard deviation of ED waiting room counts, model performance by hour, and the design of hourly and daily prediction models.

## Abbreviations

ARIMA: autoregressive integrated moving average
BiLSTM: bidirectional long short-term memory
CNN: convolutional neural network
ED: emergency department
ESI: Emergency Severity Index
FCNPlus: fully convolutional network plus
FCP: full capacity protocol
ICU: intensive care unit
IRB: Institutional Review Board
LSTM: long short-term memory
MAE: mean absolute error
ML: machine learning
MSE: mean squared error
N-BEATS: neural basis expansion analysis for interpretable time series forecasting
PFCT: patient flow coordination team
PFM: patient flow measure
ResNetPlus: residual network plus
RF: random forest
RMSE: root mean squared error
RNN: recurrent neural network
Seq2Seq: sequence to sequence learning with neural networks
TSiTPlus: time series vision transformer plus
TSTPlus: time series transformer plus
XCMPlus: explainable convolutional neural network plus
XGBoost: extreme gradient boosting

## Data Availability

The data used in this study were obtained through a formal collaboration with our partner hospital and are subject to strict confidentiality agreements, institutional review board regulations, and institutional data protection policies. Access is limited to authorized personnel for research purposes under the approved ethical framework. As such, we are unable to share the dataset outside the terms of this agreement to ensure compliance with privacy and institutional standards. However, deidentified data may be shared under a data-use agreement upon reasonable request and institutional approval. The source code and model implementation are available in our GitHub repository [71].
