# Supplementary material for: An Artificial Intelligence–Based Framework for Predicting Emergency Department Overcrowding: Development and Evaluation Study
Source: JMIR Med Inform. 2025 Sep 17;13:e73960. doi: 10.2196/73960 (PMC12489414; doi:10.2196/73960)
Supplement: Multimedia Appendix 1 [file medinform_v13i1e73960_app1.docx]

## Multimedia Appendix 2

### **Feature Engineering and Data Preprocessing**

We applied a series of feature engineering and preprocessing steps to transform raw data into structured input for Machine Learning (ML) algorithms. These steps prepared the data for modeling, as described in Equation 1 of the *Problem Modeling* section in the main manuscript. First, each of the four data sources, including Emergency Department (ED) tracking data, inpatient records, weather data, and significant event dates, were processed separately to extract meaningful features before applying preprocessing steps. All features were calculated at an hourly frequency, with each row in the dataset corresponding to one hour. A comprehensive summary of all features used in the study, including the complete list of engineered features and their descriptions, is listed as follows:

- ***Date Features:*** Temporal information for each row in the dataset was represented by breaking down each timestamp into individual components, including the calendar year, the numerical month (e.g., January as 1), the specific day within the month (1-31), the day of the week as an integer (e.g., Monday as 0 and Sunday as 6), and the hour of the day in a 24-hour format. The dataset is organized into an hourly frequency, with each row corresponding to a one-hour interval. These date features are common across all data sources.
- ***Waiting Count:*** The ED tracking dataset was filtered to include only waiting room encounters and grouped by the visit ID. For each patient-visit combination, the earliest arrival $A_{i}$ and latest departure $D_{j}$ times in the waiting rooms were identified to track patient presence. The total hourly waiting count, *N(t)*, is calculated by summing all patients present within each hourly interval starting at the hour *t*, where a patient is counted if their earliest arrival *Ai* occurs before the end of the hour *t+1* and their latest departure $D_{j}$ is at or after the start of the hour *t*, as represented in Equation 2. The indicator function $I$is equal to 1 if these conditions are met and 0 otherwise, and $n(t)$represents the total number of patients considered for hour *t* (i.e., the number of unique visits during *[t, t+1]* hourly interval). This hourly waiting count serves as the target feature for our predictive modeling in this paper.

$N(t)=\sum_{i=1}^{n(t)} I\left( A_{i}<t+1 and D_{j}\geq t \right)$ (2)

- ***Waiting Counts by ESI Levels:*** The waiting count at each hourly interval was also calculated for three categories of patients based on their ESI levels: 1-2 (very urgent), 3 (urgent), and 4-5 (non-urgent). The hourly waiting count for each group was calculated using the same method as the overall hourly waiting count, relying on arrival and departure times to track patient presence for each ESI level category.
- ***Average Waiting Time:*** Waiting times were calculated for each patient who fits the criteria for the waiting count based on their ED arrival and departure times within a specific hourly interval, ensuring an accurate measurement of time spent in the waiting room. The average waiting time for each hourly interval *[t, t+1]*, Average Waiting Time ($t$), was calculated by summing the waiting times $WT_{i}(t)$ for all waiting patients and dividing by the number of waiting patients $N(t)$ during that hour, as represented in Equation 3.

$Average Waiting Time (t)=\frac{\sum_{i=1}^{n(t)} WTi(t)}{N(t)}$ (3)

- ***Average Waiting Times by ESI Levels*:** The hourly average waiting time was calculated separately for three patient groups based on their ESI levels. The calculation followed the same approach as the overall average waiting time but was applied within each ESI level category. Specifically, the arrival and departure times of patients within each ESI group were used to determine individual waiting times, which were then averaged within that group.
- ***Treatment Count****:* The treatment count for each hourly interval was calculated using the same method as the waiting count. However, instead of considering waiting rooms, only patients in treatment rooms were counted. This was done by filtering the data to include only treatment room locations when tracking patient arrivals and departures.
- ***Average Treatment Time:*** The average treatment time was calculated similarly to the overall average waiting time but focused specifically on patients in treatment rooms.
- ***Extreme Case Indicator:*** The Extreme Case Indicator (ECI) is a binary feature used to identify periods of exceptionally high patient volume. It is assigned a value of 1 when the hourly waiting count ($WCt)$ at time $t$exceeds a statistical threshold, calculated as the mean (μ) plus two standard deviations (σ) of the waiting count distribution, as defined in Equation 4. Here, $WCt$ represents the actual number of patients waiting during a specific hourly interval. This threshold accounts for natural variations in patient volume, ensuring that only significantly high values are classified as extreme. If $WCt$ remains below this threshold, the ECI is set to 0, indicating a non-extreme period.

$ECI\left( t \right)=\left\{ \begin{aligned} 1, &if WCt\geq\mu+2\sigma\\ 0, &otherwise \end{aligned} \right.$ (4)

- ***Boarding Count:*** Boarding patients are those who have completed ED treatment, decided to be admitted, and are waiting for an inpatient bed to become available after a bed request has been made. The boarding period begins when the inpatient bed request is submitted and ends when the patient leaves the ED for the inpatient unit (marked by the ED checkout timestamp). Using these start and end times, the hourly boarding count was calculated similar to the waiting counts calculation. For instance, if a patient receives an inpatient bed request at 10:10 AM and leaves the ED for the inpatient unit at 11:15 AM, their boarding period spans from 10:10 AM to 11:15 AM. Since boarding counts are calculated on an hourly basis, this patient is counted in both the 10:00 - 11:00 AM and 11:00 - 12:00 PM intervals.
- ***Average Boarding Time:*** The boarding time for a particular boarding patient was calculated by subtracting ED checkout time from inpatient bed request time. Subsequently, the average boarding time for each hourly interval was calculated by summing individual boarding times of the patients included in the boarding count for that interval and dividing it by the boarding count.
- ***Hospital Census:*** This feature represents the total number of inpatients in the hospital for a given hourly interval, excluding patients in the ED. It is calculated hourly to reflect the occupancy of inpatient units.
- ***Weather features:*** A categorical feature representing atmospheric conditions including clear skies, clouds, rain, mist, thunderstorms, snow, drizzle, haze, fog, and smoke. This feature captures hourly weather patterns, with each hourly interval potentially having a different weather status, represented as categorical variables in the dataset. Temperature, humidity, and wind speed were considered as part of the weather features.
- ***Football Game:*** This categorical feature indicates whether a football game is occurring during a given hourly interval, based on the schedule of a major team near the hospital.
- ***Federal Holidays*:** This categorical feature indicates whether a specific day is a federal holiday, such as Christmas or Independence Day. When a federal holiday occurs, the feature is assigned the corresponding label for all 24-hourly intervals of that day in the hourly dataset.
- ***Leg generation***: Lag features were created by shifting past values of the target variable to serve as additional input features, allowing the model to capture temporal patterns. For example, a 12-hour lag feature set includes waiting counts recorded at each hour from 1 to 12 hours before the current time (i.e., values at $t-1, t-2, \ldots.,t-12$). Similarly, 24-hour and 48-hour lag sets include values from the past 24 and 48 hours, respectively. These lag intervals were selected based on dataset characteristics, as detailed in Table 1 of the main manuscript.
- ***Rolling mean calculations:*** These were applied to reduce short-term variability by averaging values over a defined window. For instance, a 4-hour waiting count rolling mean concept computes the average waiting count values over the current and the previous three hourly intervals, helping to smooth sudden fluctuations. 4 and 6 hours rolling mean windows were selected as detailed in Table 1 of the main manuscript.

After feature engineering, preprocessing steps were applied to prepare the data for model training and testing, as illustrated in Figure 1 of the main manuscript. These steps include categorizing specific features to simplify analysis and excluding certain data periods and outliers to minimize potential biases. Additionally, standardization techniques were applied to maintain consistency across numerical features. The following describes the performed preprocessing steps in detail:

- Weather data source included various weather conditions, such as clear skies, clouds, rain, mist, thunderstorms, snow, drizzle, haze, fog, and smoke. To address data sparsity and improve model performance, these conditions were grouped into five categories: Clear, Clouds (combining Clouds and Mist), Rain (combining Rain and Drizzle), Thunderstorm, and Others (including less frequent conditions like snow, haze, fog, and smoke). For example, "Clouds," "Mist," and "Drizzle" were combined due to their similar mild atmospheric effects and frequent co-occurrence, while more distinct conditions like "Rain" and "Thunderstorm" were preserved as separate categories given their stronger potential influence on patient flow. During dataset creation, two versions were generated: one retaining all ten original weather categories and another using the simplified five-category system to reduce dimensionality.
- The ED tracking data source was preprocessed to exclude instances where patients had been waiting in the waiting room for more than 9 hours. This threshold was determined in consultation with the advisory board from the partner hospital's emergency department. These cases were identified as outliers, deemed as potential data entry errors, that could affect the performance of the predictive models and were removed from the data.
- Data from January 2020 to May 2021, referred to in this study as the COVID-19 period, was excluded due to unusual patterns in patient volumes and hospital operations during the pandemic. This timeframe was characterized by significant disruptions to hospital operations and patient flow due to the pandemic. Specifically, ED and hospital volumes declined sharply, and standard care protocols were frequently altered (e.g., changes in triage procedures, elective surgery cancellations, and surge capacity adjustments). These atypical patterns deviate substantially from normal operational conditions and could impair the ability of machine learning models to learn representative trends. For example, as shown in
- Table 1 of the main manuscript, the Hospital Census, which has an average of 794, shows a significant drop to 409 on some dates during this period.
- Numerical features from all data sources were normalized using various scaling methods, such as StandardScaler [1] and MinMaxScaler [2].

### **Data Integration and Aggregation**

The data collected from various sources were processed through feature engineering and data preprocessing, then merged into a single dataset. The new dataset is referred to as *Integrated Data* in Figure 1 of the main manuscript, where each row represents an hourly interval at a frequency of 1 hour. From the integrated data, two distinct datasets were created: one for the hourly prediction model, where each row corresponds to an hourly record, and another for the daily prediction model, where each row represents the average values of the preceding 24 hours from the hourly data.

The hourly dataset contains 27,756 records, each representing a unique hourly interval of the study date range. The daily dataset, created by averaging consecutive 24-hour segments, contains 1,155 records, making it 24 times smaller than the hourly dataset. This aggregation reduces short-term fluctuations, leading to a lower standard deviation in the daily predictions, as detailed in Table 1 of the main manuscript. Table 1 provides an overview of the extracted features and their descriptive statistics for both the hourly and daily datasets after they were preprocessed.

### **Model training**

We employed eleven ML algorithms to develop the proposed hourly and daily waiting count predictions. The algorithms ranged from traditional algorithms to the state-of-the-art time series algorithms. These algorithms were grouped into four categories: traditional ML methods, recurrent neural network (RNN) based models, Convolutional Neural Network (CNN) based models, and transformer-based models. All algorithms were specifically designed to handle time series data. For CNN-based and transformer-based models, we utilized the A State-of-the-Art Deep Learning Library for Time Series and Sequential Data (TSAI) [3], an open-source framework built on PyTorch [4] and Fastai [5], specifically designed for time series tasks. The algorithms were trained using 16 different datasets, as outlined in Table S2 in Multimedia Appendix 3. During the models training, the datasets were divided into 70% training, 15% validation, and 15% testing. Hyperparameter optimization was initially performed using grid search over a predefined subset of the hyperparameter space, tuning key parameters such as learning rate, batch size, dropout rate, weight decay, the number of hidden units, activation function, and other model-specific parameters as applicable.

#### **Traditional Machine Learning Algorithms**

Random Forest (RF) [6] constructs multiple decision trees using bootstrap sampling and random feature selection. By aggregating the outputs of these trees, the model reduces variance, effectively handles high-dimensional data, and minimizes the risk of overfitting. In this study, we used scikit-learn's [7] implementation and experimented with hyperparameters such as the number of trees, tree depth, minimum samples for splits and leaf nodes, and the use of bootstrap sampling.

Extreme Gradient Boosting (XGBoost) [8] optimizes the loss function using a second-order Taylor expansion, allowing precise gradient approximation and efficient handling of missing data. Regularization techniques reduce overfitting, making it suitable for high-dimensional datasets. In this study, we used XGBoost's implementation and experimented with hyperparameters such as the number of trees, tree depth, learning rate, minimum loss reduction, minimum child weight, tree method, and dropout rate.

#### **Recurrent Neural Network Based Algorithms**

Long Short-Term Memory (LSTM) [9] is a type of RNN designed to handle long-term patterns in sequential data. It uses memory cells with input, forget, and output gates to control what information is retained, updated, or discarded over time. In this implementation, the LSTM model is constructed using the Keras functional API [10], allowing flexibility in architecture design. The model consists of three stacked LSTM layers, each returning both hidden and cell states, followed by dropout layers for regularization. The extracted LSTM features are processed through three dense layers.

Bidirectional Long Short-Term Memory (BiLSTM) [11] extends the LSTM architecture by processing input sequences in both forward and backward directions, enabling the model to effectively capture dependencies from past and future time steps. The model is constructed using the Keras functional API.

Sequence to Sequence Learning with Neural Networks (Seq2Seq) [12] is a neural network framework designed for tasks where input and output sequences differ in length or structure, such as time series forecasting and natural language processing. This model employs an encoder-decoder architecture, where the encoder processes the input sequence and generates a context representation, which is then used by the decoder to reconstruct the output sequence. In this implementation, LSTM layers are utilized within the Seq2Seq framework to effectively capture and utilize temporal dependencies.

#### **Convolutional Neural Network Based Algorithms**

Fully Convolutional Network Plus (FCNPlus) [13] builds on the foundational Fully Convolutional Networks (FCNs), originally developed for semantic segmentation in computer vision [14] and later adapted for time-series modeling [15]. FCNPlus enhances traditional architecture by incorporating residual connections to improve gradient flow and training stability. It also integrates features like dropout for regularization and batch normalization to stabilize and accelerate convergence.

Residual Network Plus (ResNetPlus) [16] builds upon the foundational Residual Network (ResNet) architecture [17], which was originally developed for image recognition in computer vision tasks. ResNet introduced the concept of residual connections to alleviate the vanishing gradient problem and facilitate the training of deep neural networks. In the context of time-series modeling, ResNetPlus adapts this approach by integrating features like separable convolutions, coordinate convolutions, and batch normalization to efficiently capture temporal dependencies and enhance representation learning.

XceptionTimePlus [18] is a specialized deep learning model designed for time series classification, inspired by the principles of the Xception [19] architecture. It extends the foundational ideas of depthwise separable convolutions, as introduced in Xception, to the domain of sequential data. The model is composed of modular XceptionBlocks, which include separable convolutions for efficient feature extraction, bottleneck layers to reduce computational cost, and residual connections to facilitate gradient flow and improve learning stability.

Explainable Convolutional Neural Network for Multivariate Time Series (XCM) [20] is a specialized deep learning architecture tailored for multivariate time series classification tasks. It leverages a hybrid structure combining 2D convolutions to extract inter-variable dependencies and 1D convolutions to capture intra-variable temporal patterns. By integrating these multi-scale features, XCM can effectively model complex temporal relationships across variables. For this study, XCMPlus [21] was used, a variant of XCM with a different backbone structure and feature extraction approach. Unlike XCM, which applies separate 2D and 1D convolutions before concatenation, XCMPlus integrates these layers in a sequential manner. It also incorporates adaptive kernel size selection and adjustments in dropout, batch normalization, and classification layers, modifying the processing flow for multivariate time series classification.

#### **Transformer Based Algorithms**

Time Series Transformer Plus (TSTPlus) [22] is a transformer-based model designed for multivariate time series tasks, inspired by Time Series Transformer (TST) [23]. It uses multi-head self-attention to capture both local and global temporal patterns and includes learnable positional encodings to handle sequence structure. The model offers configurable parameters such as attention heads, feedforward dimensions, and encoder layers, making it suitable for various applications.

Time Series Inception Transformer Plus (TSiTPlus) [24] is a time series transformer model that builds on the concepts introduced in the Vision Transformer (ViT) [25]. Inspired by ViT's success in treating image patches as sequential inputs for transformer-based processing, TSiTPlus adapts this idea for time series data. Instead of processing spatial image patches, TSiTPlus treats time series segments as input tokens, enabling it to model long-term dependencies and relationships within sequential data. TSiTPlus processes time series data by dividing it into smaller segments, similar to how Vision Transformers handle image data. The core of TSiTPlus is a self-attention-based encoder, which enables the model to capture dependencies across time steps. The encoder consists of multiple transformer layers, each containing multi-head self-attention [26], feed forward networks, layer normalization, and residual connections. While the model utilizes a multi-head attention mechanism, the exact implementation of the attention module is referenced externally. Additionally, a locality-sensitive self-attention option is available, enhancing attention efficiency for time series patterns. This approach helps the model focus on both individual segments and overall patterns, making it effective for datasets that require understanding long-term trends and dependencies.

**Table S1**. Summary of the machine learning algorithms employed, categorized by their underlying architecture.

| **Architecture** | **Abbreviation** | **Algorithm** |
| --- | --- | --- |
| Traditional Machine Learning Algorithms | RF [6] | Random Forests |
|  | XGBoost [8] | Extreme Gradient Boosting |
| Recurrent Neural Network (RNN) Based Algorithms | LSTM [9] | Long Short-Term Memory |
|  | BiLSTM [11] | Bidirectional Long Short-Term Memory |
|  | Seq2Seq [12] | Sequence to Sequence Learning with Neural Networks |
| Convolutional Neural Network (CNN) Based Algorithms | FCNPlus [13] | Fully Convolutional Network Plus |
|  | ResNetPlus [16] | Residual Networks Plus |
|  | XceptionTimePlus [18] | Xception Time Plus |
|  | XCMPlus [21] | Explainable Convolutional Neural Network Plus |
| Transformer Based Algorithms | TSTPlus [22] | Time Series Transformer Plus |
|  | TSiTPlus [24] | Time Series Vision Transformer Plus |

### **Model evaluation**

To evaluate the proposed models, they were tested on unseen data. Four standard evaluation metrics were used, which are as follows:

- **Mean Absolute Error (MAE**): It measures the average absolute difference between predicted and actual values. It provides a straightforward interpretation of prediction error, with lower MAE values indicating better predictive performance.
- **Mean Squared Error (MSE):** It quantifies the average squared differences between predicted and actual values. This metric is sensitive to large errors, making it useful for highlighting significant deviations in predictions.
- **Root Mean Squared Error (RMSE):** It is the square root of MSE, representing prediction errors on the same scale as the target variable. It provides a more interpretable measure of error magnitude while retaining sensitivity to large errors.
- **Coefficient of Determination (R²):** It evaluates the proportion of variance in the target variable that is explained by the model. Values closer to 1 indicate that the model explains most of the variability in the target variable, while a value **closer to 0** suggests that the model has little explanatory power.

The models are ranked based on their MAE values, as MAE provides a straightforward interpretation of the average error, making it easier to understand the practical implications of the predictions. Unlike MSE, where improvements can result in larger reductions due to squared differences, MAE decreases more gradually with improvements. Therefore, even small reductions in MAE are significant in this study, as they reflect meaningful enhancements in predictive performance.

As an additional evaluation, we conducted an Extreme Case Analysis focused on rare periods of high patient volume. Three severity levels were defined based on waiting count thresholds: Extreme (>27.88, mean + 1σ; 5,097 hourly intervals), Very Extreme (>37.65, mean + 2σ; 862 intervals), and Highly Extreme (>47.42, mean + 3σ; 64 intervals). This analysis assesses the model’s reliability under critical crowding conditions. Figure S1 in Multimedia Appendix 1 presents the descriptive statistics and threshold distributions.

To further evaluate the model, its performance was analyzed across each hour of the day over a 24-hour period, providing a detailed view of how prediction accuracy fluctuates throughout the day. This analysis provides insights into the model's performance at different times of the day, correlating with patient volume trends in the ED waiting room. Figure S2 in Multimedia Appendix 1 summarizes the mean and standard deviation of hourly waiting counts. The highest average occurs at 7 PM (mean: 26.23, SD: 9.06), indicating peak activity, while the lowest is at 6 AM (mean: 6.97, SD: 4.31), reflecting a lull in ED volume.

**References**

1. StandardScaler. Scikit-learn; [cited 2025]; Available from: https://scikit-learn.org/stable/modules/generated/sklearn.preprocessing.StandardScaler.html.

2. MinMaxScaler. Scikit-learn; [cited 2025]; Available from: https://scikit-learn.org/stable/modules/generated/sklearn.preprocessing.MinMaxScaler.html.

3. Oguiza I. tsai - A state-of-the-art deep learning library for time series and sequential data. Github2023 [cited 2025]; Available from: https://github.com/timeseriesAI/tsai.

4. Paszke A, Gross S, Massa F, Lerer A, Bradbury J, Chanan G, et al. Pytorch: An imperative style, high-performance deep learning library. Advances in neural information processing systems. 2019;32.

5. Howard J, Gugger S. Fastai: a layered API for deep learning. Information. 2020;11(2):108.

6. Breiman L. Random forests. Machine learning. 2001;45:5-32.

7. Pedregosa F, Varoquaux G, Gramfort A, Michel V, Thirion B, Grisel O, et al. Scikit-learn: Machine learning in Python. the Journal of machine Learning research. 2011;12:2825-30.

8. Chen T, He T, Benesty M, Khotilovich V, Tang Y, Cho H, et al. Xgboost: extreme gradient boosting. R package version 04-2. 2015;1(4):1-4.

9. Hochreiter S, Schmidhuber J. Long short-term memory. Neural computation. 1997;9(8):1735-80.

10. Chollet F. Keras. 2015 [cited 2025]; Available from: https://keras.io/.

11. Schuster M, Paliwal KK. Bidirectional recurrent neural networks. IEEE transactions on Signal Processing. 1997;45(11):2673-81.

12. Sutskever I, Vinyals O, Le QV. Sequence to sequence learning with neural networks. Advances in neural information processing systems. 2014;27.

13. Oguiza I. FCNPlus. Github; 2023 [cited 2025]; Available from: https://timeseriesai.github.io/tsai/models.fcnplus.html.

14. Long J, Shelhamer E, Darrell T, editors. Fully convolutional networks for semantic segmentation. Proceedings of the IEEE conference on computer vision and pattern recognition; 2015.

15. Wang Z, Yan W, Oates T, editors. Time series classification from scratch with deep neural networks: A strong baseline. 2017 International joint conference on neural networks (IJCNN); 2017: IEEE.

16. Oguiza I. ResNetPlus. Github; 2023 [cited 2025]; Available from: https://timeseriesai.github.io/tsai/models.resnetplus.html.

17. He K, Zhang X, Ren S, Sun J, editors. Deep residual learning for image recognition. Proceedings of the IEEE conference on computer vision and pattern recognition; 2016.

18. Oguiza I. XceptionTimePlus. Github; 2023 [cited 2025]; Available from: https://timeseriesai.github.io/tsai/models.xceptiontimeplus.html.

19. Chollet F, editor. Xception: Deep learning with depthwise separable convolutions. Proceedings of the IEEE conference on computer vision and pattern recognition; 2017.

20. Fauvel K, Lin T, Masson V, Fromont É, Termier A. Xcm: An explainable convolutional neural network for multivariate time series classification. Mathematics. 2021;9(23):3137.

21. Oguiza I. XCMPlus. Github2023 [cited 2025]; Available from: https://timeseriesai.github.io/tsai/models.xcmplus.html.

22. Oguiza I. TSTPlus. Github; 2023 [cited 2025]; Available from: https://timeseriesai.github.io/tsai/models.tstplus.html.

23. Zerveas G, Jayaraman S, Patel D, Bhamidipaty A, Eickhoff C, editors. A transformer-based framework for multivariate time series representation learning. Proceedings of the 27th ACM SIGKDD conference on knowledge discovery & data mining; 2021.

24. Oguiza I. TSiT. Github; 2023 [cited 2025]; Available from: https://timeseriesai.github.io/tsai/models.tsitplus.html.

25. Dosovitskiy A, Beyer L, Kolesnikov A, Weissenborn D, Zhai X, Unterthiner T, et al. An image is worth 16x16 words: Transformers for image recognition at scale. arXiv preprint arXiv:201011929. 2020.

26. Vaswani A, Shazeer N, Parmar N, Uszkoreit J, Jones L, Gomez AN, et al. Attention is all you need. Advances in neural information processing systems. 2017;30.
