# Supplementary material for: An Artificial Intelligence–Based Framework for Predicting Emergency Department Overcrowding: Development and Evaluation Study
Source: JMIR Med Inform. 2025 Sep 17;13:e73960. doi: 10.2196/73960 (PMC12489414; doi:10.2196/73960)
Supplement: Multimedia Appendix 2 [file medinform_v13i1e73960_app2.docx]

## Multimedia Appendix 3

**Table S2.** Features and Their Combinations for Dataset Variants.

| **Data Sources and Scaling** | **Features** | **Lags and  Rolling Mean** | **DS 0** | **DS 1** | **DS 2** | **DS 3** | **DS 4** | **DS 5** | **DS 6** | **DS 7** | **DS 8** | **DS 9** | **DS 10** | **DS 11** | **DS 12** | **DS 13** | **DS 14** | **DS 15** |
| --- | --- | --- | --- | --- | --- | --- | --- | --- | --- | --- | --- | --- | --- | --- | --- | --- | --- | --- |
| ED Tracking | Waiting Count | Lags (W=24) | X | X | X | X | X | X | X | X | X | X | X | X | X | X |  | X |
|  |  | Lags (W=48) |  |  |  |  |  |  |  |  |  |  |  |  |  |  | X |  |
|  |  | Rolling Mean (W=4) |  |  |  |  |  |  |  |  |  |  | X | X | X | X | X | X |
|  |  | Rolling Mean (W=6) |  |  |  |  |  |  |  |  |  | X |  |  |  |  |  |  |
|  | Average Waiting Time | No Lags |  |  |  |  |  |  |  |  | X | X | X | X | X |  | X | X |
|  |  | Lags (W=24) |  |  |  |  |  |  |  |  |  |  |  |  |  | X |  |  |
|  |  | Rolling Mean (W=4) |  |  |  |  |  |  |  |  |  |  |  |  |  | X |  |  |
|  | Treatment Count | No Lags |  |  |  |  |  |  |  | X | X | X | X | X | X |  | X | X |
|  |  | Lags (W=24) |  |  |  |  |  |  |  |  |  |  |  |  |  | X |  |  |
|  |  | Rolling Mean (W=4) |  |  |  |  |  |  |  |  |  |  |  |  |  | X |  |  |
|  | Boarding Count | No Lags |  |  |  |  |  |  |  | X | X | X | X | X | X |  | X | X |
|  |  | Lags (W=24) |  |  |  |  |  |  |  |  |  |  |  |  |  | X |  |  |
|  |  | Rolling Mean (W=4) |  |  |  |  |  |  |  |  |  |  |  |  |  | X |  |  |
|  | Waiting Count by ESI Levels (3)^a^ |  |  |  |  |  |  | X | X | X | X | X | X | X | X | X | X | X |
|  | Average Waiting Time by ESI Levels (3)^a^ |  |  |  |  |  |  |  |  |  | X | X | X | X | X | X | X | X |
|  | Average Treatment Time |  |  |  |  |  |  |  |  | X | X | X | X | X | X | X | X | X |
|  | Average Boarding Time |  |  |  |  |  |  |  |  | X | X | X | X | X | X | X | X | X |
|  | Extreme Case Indicator |  | X | X | X | X | X | X | X | X | X | X | X | X | X | X | X | X |
|  | Year, Month, Day of the Month, Day of the Week, Hour (5)^a^ |  | X | X | X | X | X | X | X | X | X | X | X | X | X | X | X | X |
| Weather | Temperature | No Lags |  |  |  | X |  |  |  |  |  |  |  |  |  |  |  | X |
|  |  | Lags (W=24) |  |  |  |  | X |  |  |  |  |  |  |  |  | X |  |  |
|  | Wind speed |  |  |  |  | X | X |  |  |  |  |  |  |  |  |  |  | X |
|  | Humidity |  |  |  |  | X | X |  |  |  |  |  |  |  |  |  |  | X |
|  | Weather Status (5 Categories)^a^ |  |  |  |  |  |  | X | X | X | X | X | X | X | X | X | X | X |
|  | Weather Status (10 Categories)^a^ |  |  |  |  | X | X |  |  |  |  |  |  |  |  |  |  |  |
| Inpatient | Hospital Census | No Lags |  |  | X | X | X | X | X | X | X | X | X |  |  |  |  |  |
|  |  | Lags (W=24) |  |  |  |  |  |  |  |  |  |  |  | X | X | X | X | X |
|  |  | Rolling Mean (W=6) |  |  |  |  |  |  |  |  |  |  |  | X |  | X |  |  |
| Significant Dates | Federal Holidays |  |  |  |  |  |  |  | X | X | X | X | X | X | X | X | X | X |
|  | Football Game |  |  |  |  |  |  |  | X |  |  |  |  |  |  |  |  | X |
|  | COVID-19 | Exclude between 01-01-2020  and  05-01-2021 | X | X | X | X | X | X | X | X | X | X | X | X | X | X | X | X |
| Scaling | MinMax |  | X |  |  |  |  |  |  |  |  |  |  |  |  |  |  |  |
|  | Z-score |  |  | X | X | X | X | X | X | X | X | X | X | X | X | X | X | X |
| Number of Features |  | | 30 | 30 | 31 | 44 | 67 | 39 | 41 | 44 | 48 | 49 | 49 | 73 | 72 | 169 | 96 | 76 |

a: indicates the number of features24
