# Supplementary material for: An Artificial Intelligence–Based Framework for Predicting Emergency Department Overcrowding: Development and Evaluation Study
Source: JMIR Med Inform. 2025 Sep 17;13:e73960. doi: 10.2196/73960 (PMC12489414; doi:10.2196/73960)
Supplement: Multimedia Appendix 3 [file medinform_v13i1e73960_app3.docx]

## Multimedia Appendix 1


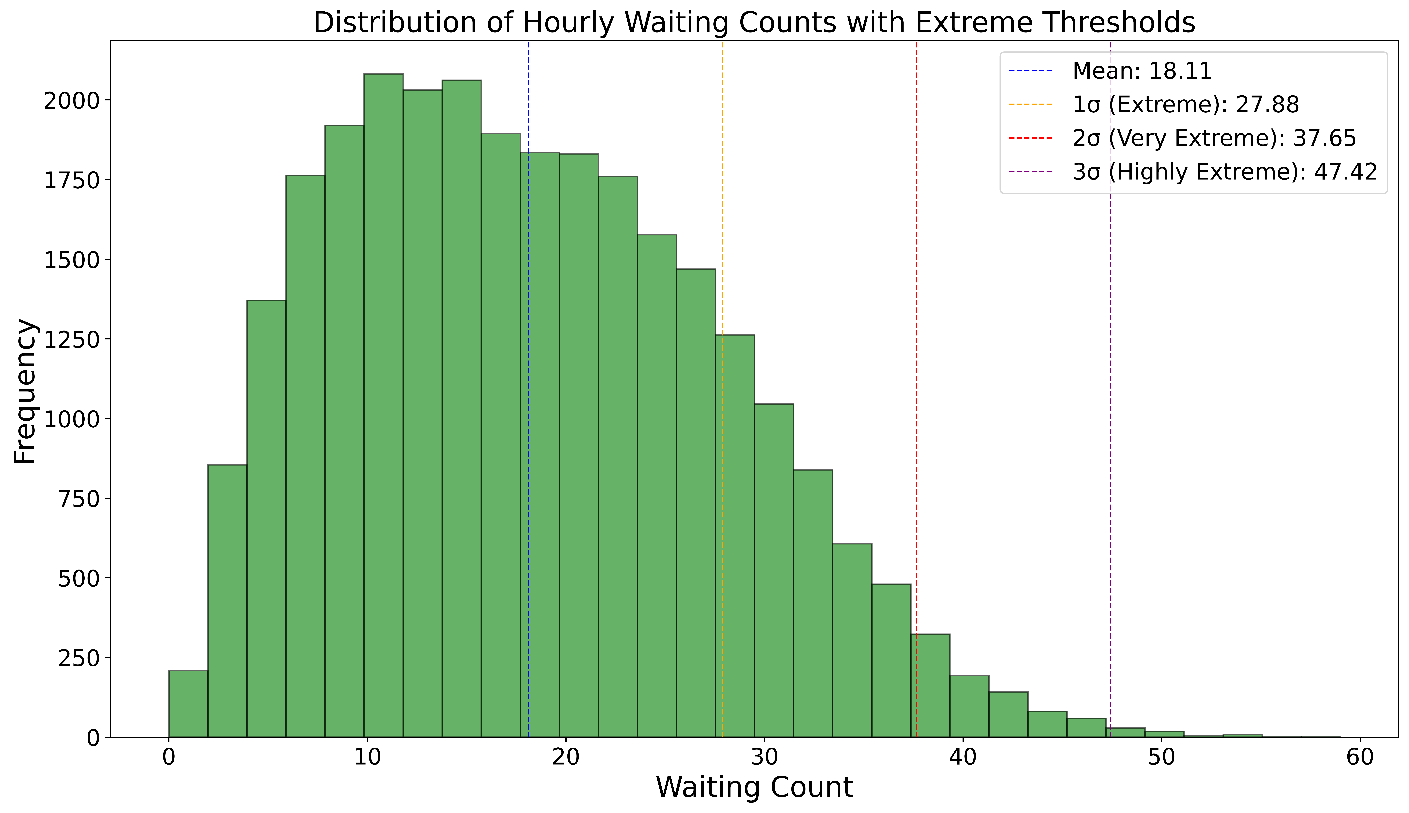


**Figure S1.** Distribution of Hourly Waiting Counts with Extreme Thresholds


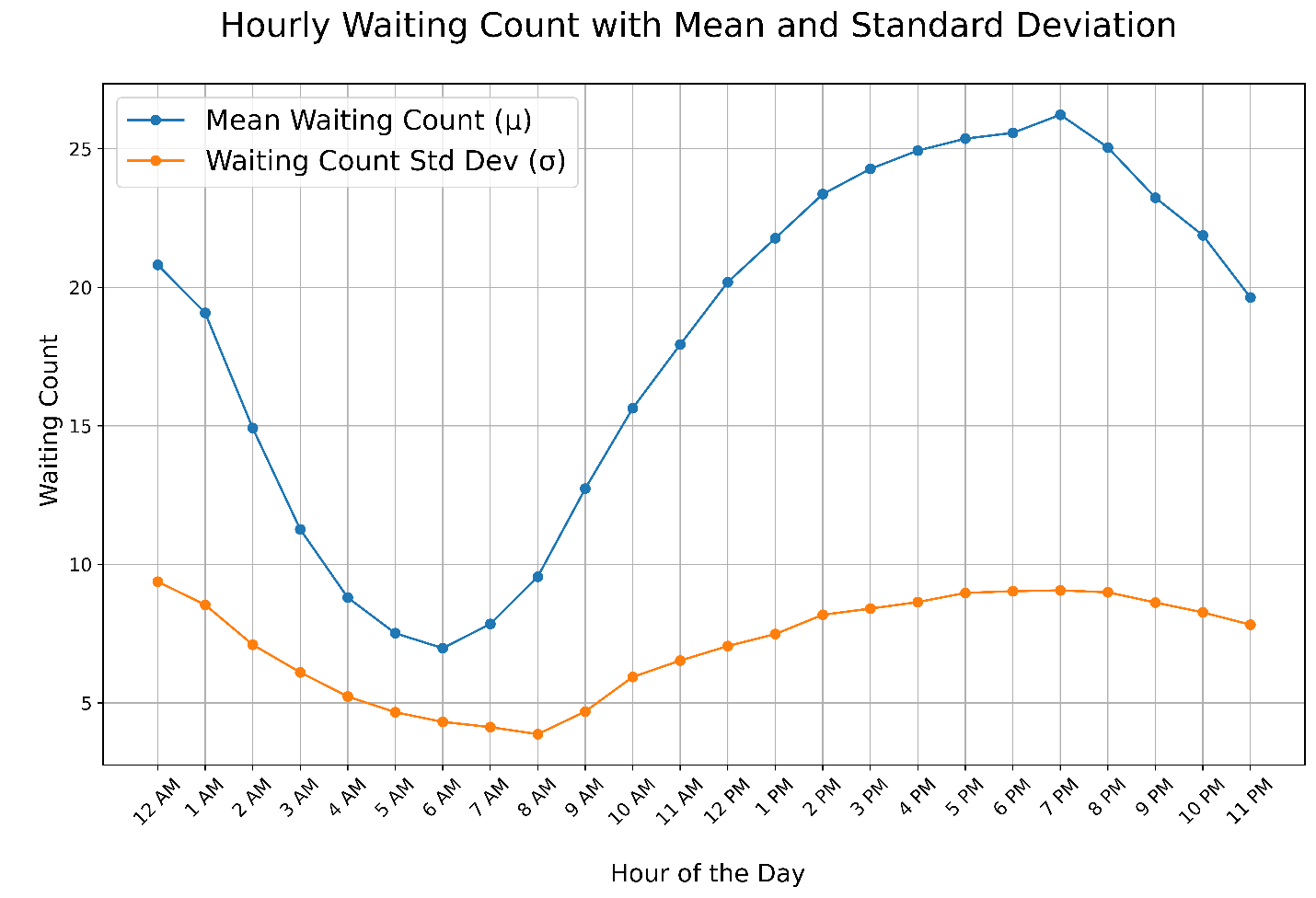


**Figure S2.** Mean and standard deviation of hourly waiting counts in the emergency department waiting room across the Hour-of-Day (12 AM – 11 PM), illustrating variations in patient volume and fluctuations throughout the day.


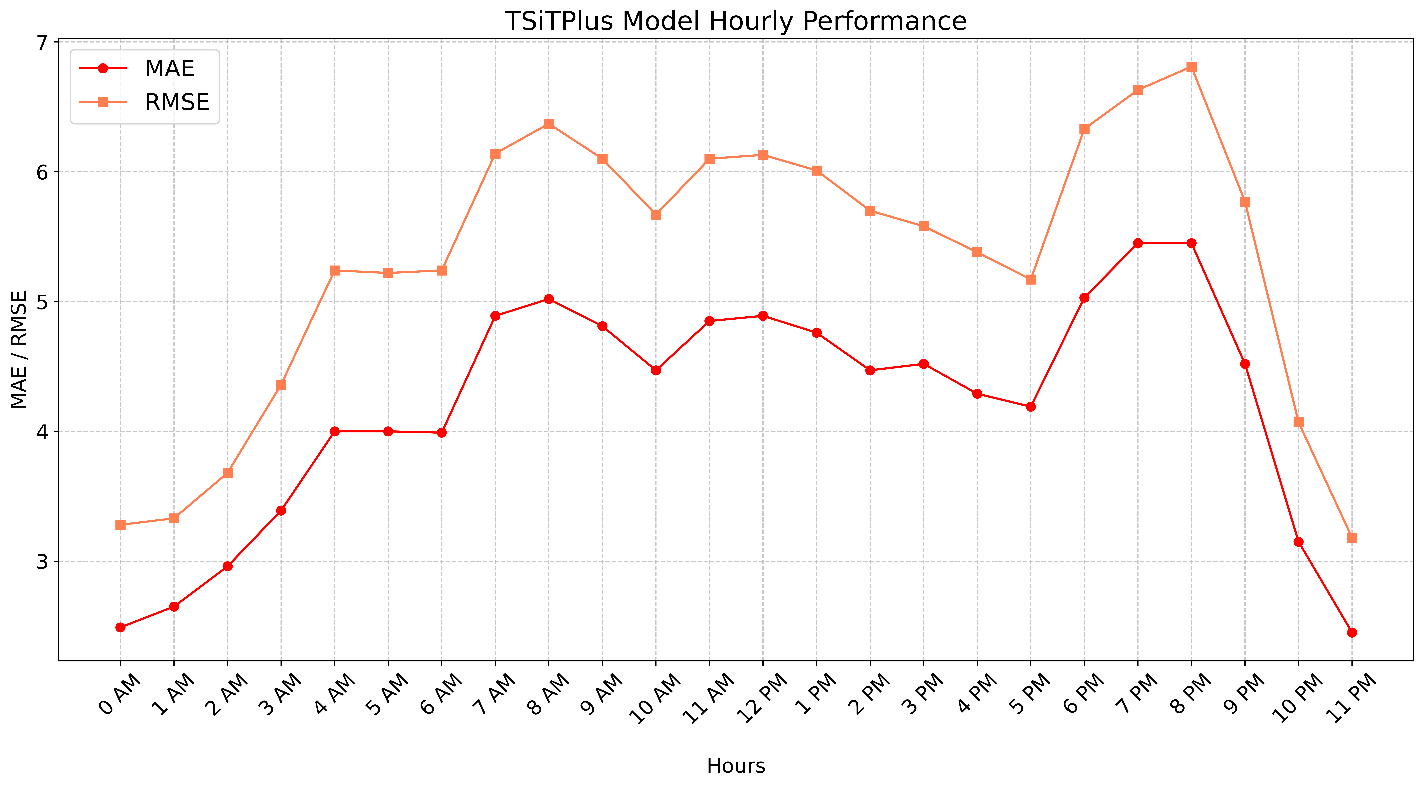


**Figure S3.** Hour-of-Day Analysis of TSiTPlus Model Performance Using MAE and RMSE


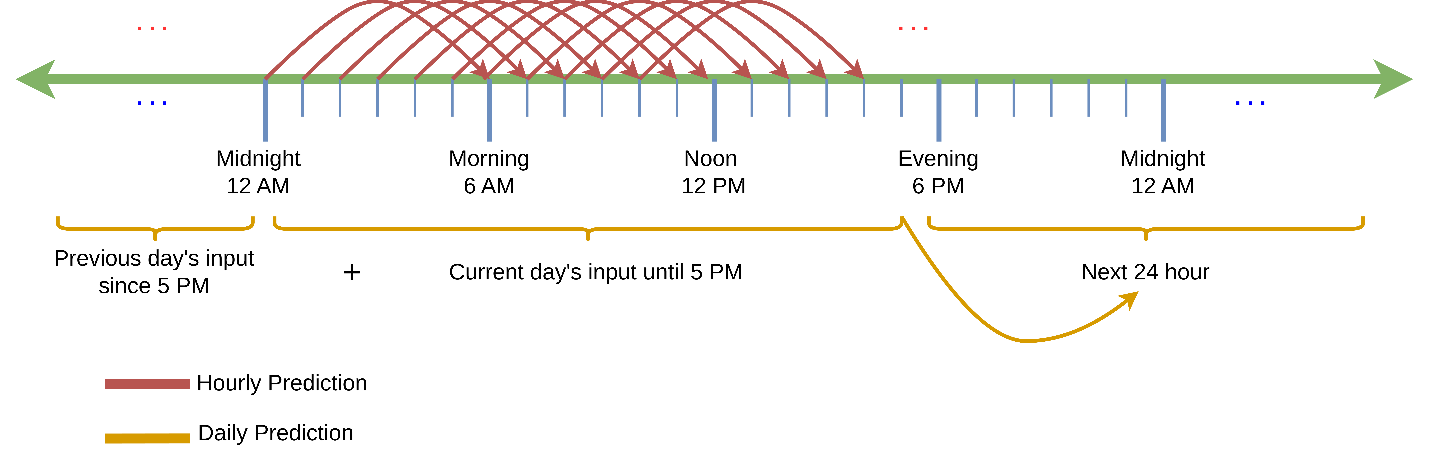


**Figure S4.** Illustration of Hourly and Daily Prediction Models for ED Waiting Count Forecasting.
